# Prussian Blue Analogues as Anode Materials for Battery Applications: Complexities and Horizons

**DOI:** 10.1021/acs.chemmater.5c00213

**Published:** 2025-06-03

**Authors:** Mario Palacios-Corella, Igor Echevarria, Carla Santana Santos, Wolfgang Schuhmann, Edgar Ventosa, Maria Ibáñez

**Affiliations:** † Institute of Science and Technology Austria (ISTA), Am Campus 1, Klosterneuburg, 3400, Austria; ‡ Analytical Chemistry -Center for Electrochemical Sciences (CES), Faculty of Chemistry and Biochemistry, Ruhr University Bochum, Universitätsstraße. 150, Bochum, D-44780, Germany; § International Research Center in Critical Raw Materials-ICCRAM, Universidad de Burgos, Plaza Misael Bañuelos s/n, E-09001, Burgos, Spain; ∥ Departamento de Química, Facultad de Ciencias, Universidad de Burgos, Pza. Misael Bañuelos s/n, E-09001, Burgos, Spain

## Abstract

Prussian blue (PB) and Prussian blue analogues (PBAs) are a class of porous materials composed of transition metal cations, cyanide ligands, and alkali metal cations. Their ability to intercalate and deintercalate ions within their framework pores, coupled with the adaptability of their crystal structure to electrochemical changes, underpins their success in battery applications. PBAs with Fe or Co as the active site exhibit high redox potentials (vs SHE) and have been extensively explored as cathode materials, with well-documented chemistry, crystal structures, and electrochemical properties. In contrast, PBAs with Cr or Mn as the active site display lower redox potentials and remain significantly underexplored as anode materials. This gap has led to fewer reported compounds and a less comprehensive understanding of their structural and electrochemical behavior, leaving the field relatively opaque. In this perspective, we comprehensively analyze the challenges involved in producing and employing PBAs with low redox potentials as active battery materials. Conversely, we propose numerous horizons and ask fundamental questions that should pave the way for future research to advance the field.

## Graphical abstract

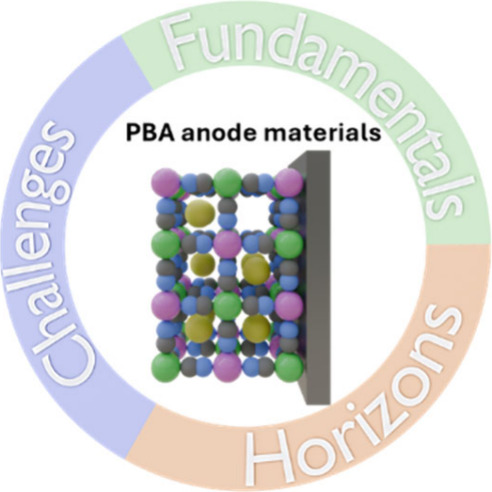

## 1 Introduction

Prussian blue (PB) and its analogues (PBAs) are a class of porous coordination polymers formed by the interconnection of cyanide ligands and metallic centers. The name “Prussian blue” originates from the deep blue pigment first synthesized in 1706 by a Prussian paint maker and an alchemist.^1^ This iconic color emerged accidentally when iron­(II) sulfate (FeSO_4_) was mixed with blood-contaminated potash, producing Fe^III^[Fe^II^(CN)_6_]_0.75_.

Beyond their historical role as dyes, PB and PBAs have captivated the scientific community^2^ and gained significant attention across various scientific fields, particularly in energy storage, where they have primarily been used as cathode materials, while their applicability as anode materials has been explored to a lesser extent. This perspective delves into PBAs with low redox potentials (below 0 V vs SHE) as anode materials, addressing the multiple challenges they face before being implemented in battery applications.

The discussion takes a critical approach, acknowledging the limited applicability of these materials in consumer electronics, especially in nonaqueous Li- and Na-ion batteries, where both economic and practical constraints make their use unfeasible. These limitations stem from their relatively high cost, poor cycling stability during Li^+^ insertion/deinsertion,^3^ and low specific capacity (60 mAh·g^–1^),^4^ compared to leading alternatives: graphite (∼372 mAh·g^–1^),^5^ lithium metal (3860 mAh·g^–1^),^6^ and lithium metal oxides like LiCoO_2_ and LiNiO_2_ (300 mAh·g^–1^).^7^ In the context of nonaqueous Na-ion batteries, hard carbons (HCs, ∼ 300 mAh·g^–1^)^8^ offer a far more cost-effective solution, further diminishing the incentive to pursue PBAs in this space.

Despite these drawbacks in nonaqueous batteries, PBAs show significant promise in alternative energy storage systems, particularly as anode materials for intercalation-based aqueous Na-ion and K-ion batteries in stationary applications.^9^ In these contexts, where cost, size, and volume constraints are less restrictive than in portable electronics, and recyclability and energy efficiency are vital, high-performance anodes remain scarce.^10–12^ For example, state-of-the-art HCs, while highly effective in nonaqueous Na-ion systems, are unsuitable in aqueous environments due to the Na^+^ intercalation potential exceeding the hydrogen evolution reaction (HER) threshold.^13^ As a result, Ti-based NASICON-type materials such as NaTi_2_(PO_4_)_3_ (133 mAh·g^–1^)^14^ have emerged as the only viable option for aqueous Na-ion anodes, though they also present limitations in terms of degradation,^15^ low conductivity,^16^ and Coulombic efficiency.^4^ A similar scenario exists for aqueous K-ion batteries, where KTi_2_(PO_4_)_3_ (58 mAh g^–1^)^17^ is among the few usable anode materials.

Given the limited availability of suitable anode materials for aqueous batteries, PBAs have emerged as promising candidates for use in aqueous Na-ion and K-ion systems. Their appeal lies in a combination of unique features, including an open-framework structure, tunable composition, exceptional ion reversibility without structural collapse, and cost-effective synthesis.^9^ In the following section, we explore these distinctive characteristics in depth, examining their fundamental origins and implications for practical applications.

## 2 Understanding PBAs

### 2.1 Composition and Structural Features of PBAs

PBAs are typically represented by the formula A_*x*_T­[R­(CN)_6_]_1_-_*y*_·wH_2_O.^18^ In these materials, the composition comprises alkali metal ions (A_*x*_), transition metal cations (T), hexacyanometallate complexes ([R­(CN)_6_]^n‑^), hexacyanometallate vacancies ([R­(CN)_6_]_*y*_), and water molecules (wH_2_O), which intricately shape the PBA crystal lattice.^19^

Generally, as-synthesized PBAs with a low alkali metal content and a composition of A_*x*_T­[R­(CN)_6_]_1_-_*y*_·wH_2_O (0 ≤ *x* ≤ 1) adopt a cubic lattice structure.^20^ Within this arrangement, T and R transition metals occupy the vertices of the cube and are octahedrally coordinated by asymmetric cyanide ligands, forming an infinite assembly of T­[R­(CN)_6_]^n‑^ (0 ≤ *n* ≤ 1) metal coordination complexes (Figure 1a). This cubic configuration creates pores or cavities where counterions such as sodium or potassium are hosted to balance the negative charges from the T­[R­(CN)_6_]^n‑^ coordination complexes, resulting in neutral coordination polymers.

**Figure 1:**
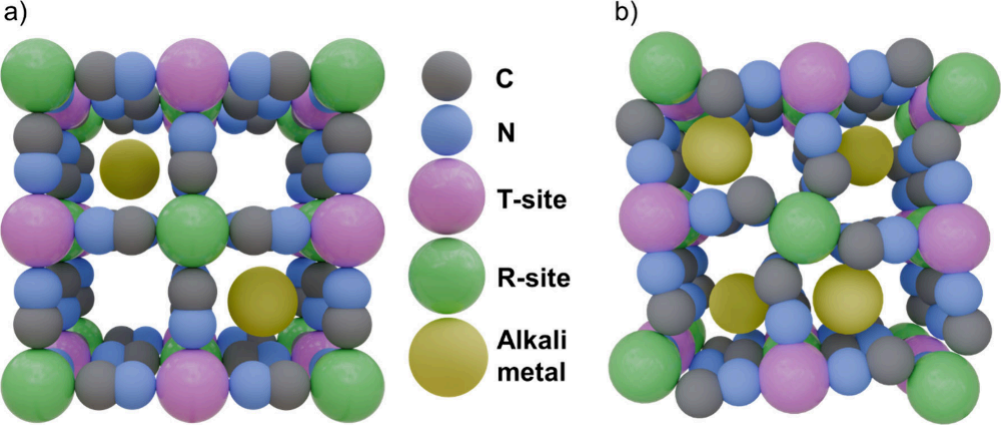
(a) Cubic lattice for PBA with one alkali metal ion per formula unit; (b) Distorted rhombohedral lattice with two alkali metal ions per formula unit.

In contrast, PBAs with a higher alkali metal content and a composition A_*x*_T­[R­(CN)_6_]_1_-_*y*_·wH_2_O (*x* > 1) often exhibit significant lattice distortions to accommodate the increased number of alkali metals, leading to rhombohedral, tetragonal, or monoclinic phases (Figure 1b).^20^ However, distortions can also arise in PBAs with lower alkali metal content due to the Jahn–Teller effect in octahedrally coordinated transition metals with d^4^ high spin and d^7^ low spin configurations (discussed in detail in the *Asymmetry of the CN* ^*–*^ *Ligands* subsubsection).^21^

Furthermore, the electrochemically active nature of PBAs, combined with their ion-intercalation capability, makes them structurally dynamic systems due to their open framework characteristics. Consequently, their crystalline structure often undergoes changes during electrochemical cycling.^9^

Overall, four key elements underpin the unique properties of PBAs, conquering high interest as active materials for batteries: (i) the asymmetry of the CN^–^ ligand, (ii) the insertion and deinsertion of alkali metal ions, and (iii) the coexistence of [R­(CN)_6_]_*y*_ vacancies, and (iv) structural water molecules. The following subsubsections present deeper insights into these four key elements and their repercussion in the PBA framework.

#### 2.1.1 CN^–^ Ligands

The asymmetric nature of the CN^–^ bridges plays a pivotal role in the structure of the PBA and regulates the electrochemistry of the materials. Typically, the “R” transition metal (also known as R-site cation) from the precursor hexacyanometallate salt, [R^II/III^(CN)_6_]^n‑^, is coordinated by carbon atoms in the final PBA, while the “T” transition metal (also known as T-site cation) is octahedrally coordinated by nitrogen atoms.^20^ Since most of the electronic density in the CN^–^ ligand is concentrated near the carbon atom, it acts as a stronger field ligand compared to the nitrogen atom, which acts as a weaker field ligand, despite the latter being more electronegative.^22^

On the basis of the crystal field theory, in octahedrally coordinated transition metals, this difference in ligand field strength can lead to two different spin states for a given electronic configuration depending on the splitting between t_2g_ and e_g_ orbitals.^23^ Thus, strong field ligands lead to large splitting between t_2g_ and e_g_ orbitals, giving rise to ground low spin states. Conversely, weaker field ligands lead to smaller splitting between t_2g_ and e_g_ orbitals, giving rise to ground high spin states.

When the ground spin state of the electronic configuration does not exhibit degeneracy, the R-C_6_ or T-N_6_ octahedra retain their original shape (Figure 2a). Conversely, in cases where the ground spin state of the electronic configuration exhibits degeneracy (i.e., unpaired electrons can occupy other orbitals of the same energy), significant distortions in the arrangement of the orbitals occur to eliminate this degeneracy (Figure 2b). This phenomenon, known as the Jahn–Teller effect, leads to distortions in the shape of the R-C_6_ or T-N_6_ octahedra and, consequently, in the structure of the PBA.^24^ These distortions are particularly pronounced for degenerate states in the e_g_ orbitals, such as in high spin Mn^III^ (d^4^ electronic configuration) and low spin Co^II^ (d^7^ electronic configuration).^25^ Smaller distortions also appear for degenerate states in the t_2g_ orbitals, such as in low spin Mn^II^ (d^5^ electronic configuration), high spin Fe^II^ and high spin Co^II^.^25^

**Figure 2:**
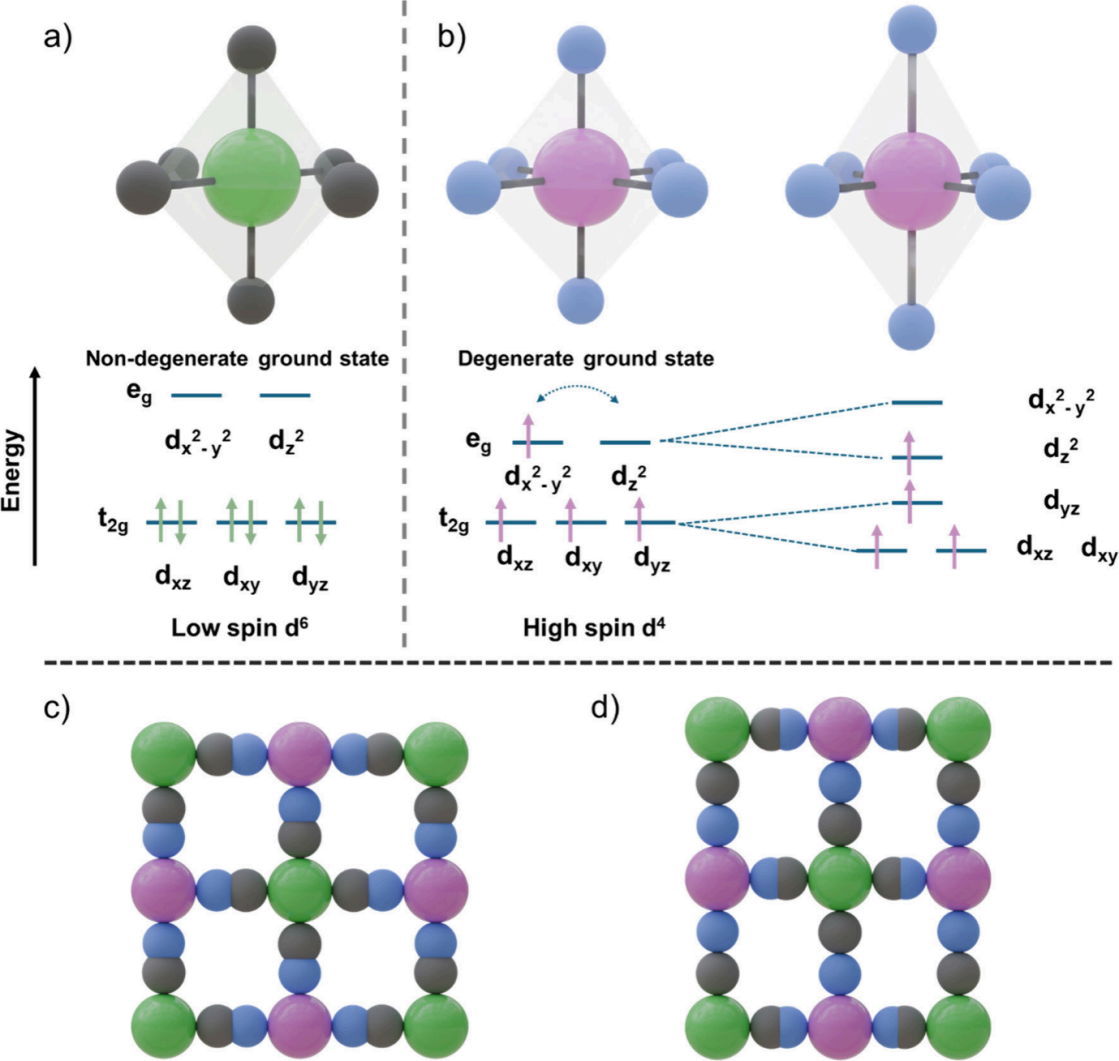
(a) Energy splitting of t_2g_ and e_g_ orbitals for octahedrally coordinated transition metals with d^6^ low spin configuration; (b) d^4^ high spin configuration; (c) nondistorted cubic structure found in Fe^III^[Fe^II^(CN)_6_]_0.75_; (d) Jahn–Teller elongated tetragonal structure found in Mn^III^[Fe^III^(CN)_6_]. The color code used is as follows: R-site Fe^III^ (green), T-site Mn^III^ (pink), C (gray), N (blue).

Thus, PBAs containing Mn^III^–N_6_ or Co^II^–C_6_ units often experience notable distortions in their crystalline structure, which can affect alkali metal ion insertion and deinsertion during electrochemical cycling. For example, in the octahedra formed by the Fe^II^–C_6_ units found in PB, Fe^III^[Fe^II^(CN)_6_]_0.75_, the Fe^II^ ions adopt a low-spin ground state, with six electrons fully occupying the t_2_g orbitals. Conversely, in the Fe^III^–N_6_ octahedra, the Fe^III^ (d^5^ electronic configuration) adopts a high spin ground state, with three electrons in t_2_g and two in e_g_ orbitals. Since neither of these two ground states exhibits degeneracy, both the Fe^II^–C_6_ and Fe^III^–N_6_ octahedra shapes are retained without distortion, and PB displays a cubic structure (Figure 2c).^26^

In contrast, in the octahedra formed by the Fe^III^–C_6_ units found in Mn^III^[Fe^III^(CN)_6_], the Fe^III^ adopts a low spin ground state where five electrons partially fill the t_2g_ orbitals. Conversely, in the Mn^III^–N_6_ octahedra, the Mn^III^ (d^4^ electronic configuration) adopts a high spin ground state where three electrons fill the t_2g_ orbitals and one electron fills the e_g_ orbitals. In this case, both ground states exhibit degeneracy. However, degenerate states associated with e_g_ orbitals cause stronger distortions compared to those associated with t_2g_ orbitals. Hence, distortions generated by the Mn^III^–N_6_ units lead to a final tetragonal crystalline structure in Mn^III^[Fe^III^(CN)_6_] (Figure 2d).^27^

Beyond the structural influence, the asymmetric electronic density distribution in CN^–^ ligands has a major impact in the electrochemical properties of PBAs. Typically, redox processes occur at the R-site cation (active metal),^9^ while the T-site cation (inactive metal) remains electrochemically inactive due to its redox potentials being either too high or too low,^28,29^ a condition far from the common work conditions. However, the T-site cation can become electrochemically active when occupied by Mn, Fe, or Co, increasing the specific capacity of the PBA.^30^ In these cases, though, the material’s cycling stability may be compromised, not only due to distortions arising from the oxidation or reduction of the R-site cation but also because Mn, Fe, or Co can introduce additional distortions, such as Jahn–Teller distortions, which may cause crystal phase transformations.^31–33^

#### 2.1.2 Alkali Metal Ions

Alkali metal ions play major roles in the composition, structure, and electrochemistry of PBAs. Primarily, alkali metals act as counterions and compensate the negative charges generated upon the formation of T­[R­(CN)_6_]^n‑^ coordination complexes, allowing to precipitate neutral coordination polymers.^19^ Among alkali metal cations, Na^+^ and K^+^ are the most used alkali metal counterions, although Cs^+^ has also been explored to a lesser extent.^34,35^

Second, the size and number of alkali metals together with [R­(CN)_6_]_*y*_ vacancies coregulate the crystal structure of PBAs. Generally, most PBAs that combine divalent/trivalent and trivalent/trivalent transition metal cations (e.g., KNi^II^[Fe^III^(CN)_6_] or Fe^III^[Fe^III^(CN)_6_] respectively) present a cubic structure and can host up to one alkali metal per formula unit.^20^ On the other end, PBAs that combine exclusively divalent metal cations (e.g., K_2_Mn^II^[Mn^II^(CN)_6_] or Na_2_Co^II^[Fe^II^(CN)_6_]) can incorporate two alkali metals in the framework. Although it depends on the size and number, the incorporation of two alkali metals often leads to significant distortions in the cubic phase to accommodate them, resulting in a rhombohedral, tetragonal, or monoclinic phase.^20^ Nonetheless, the presence of [R­(CN)_6_]_*y*_ vacancies can minimize distortions in the crystal structure,^36^ compensating the distortions of alkali metals insertion (discussed further in *[R­(CN)*_*6*_ *]­y Vacancies and Water Molecules* subsubsection).

Third, during the electrochemical cycling of PBAs, insertion and deinsertion of alkali ions in the PBA framework take place to compensate for the reduction and oxidation processes of R- and T-site cations. The radius of the alkali ion influences the free energy of the insertion reaction, generally following the trend: the larger the ionic radius, the higher the reaction potential. For instance, the insertion potential for monovalent alkali metals in KCu^II^[Fe^III^(CN)_6_] follows the order K^+^ > Na^+^ > Li^+^ (0.93, 0.8, and 0.7 V versus SHE, respectively).^37^

It should be noted that the electrochemical insertion or deinsertion of alkali metals leads to changes in the composition that can result in crystallographic phase transitions (Figure 3).^38^ For example, as-synthesized Na_1.96_Mn^II^[Mn^II^(CN)_6_]_0.99_ has a monoclinic crystal structure. However, upon oxidation of both manganese centers, both sodium ions are deinserted, resulting in a compound with a cubic structure and composition Mn^III^[Mn^III^(CN)_6_]_0.99_.^39^ Moreover, although electrochemically induced phase transitions can be reversible, the repeated insertion and deinsertion of alkali metals can generate significant strain in the CN bonds. In turn, this strain can lead to the dissolution of T and R-site cations, consequently causing changes in the capacity of inserting cations in the framework (capacity fading) and reducing the cyclability.^40,41^

**Figure 3:**
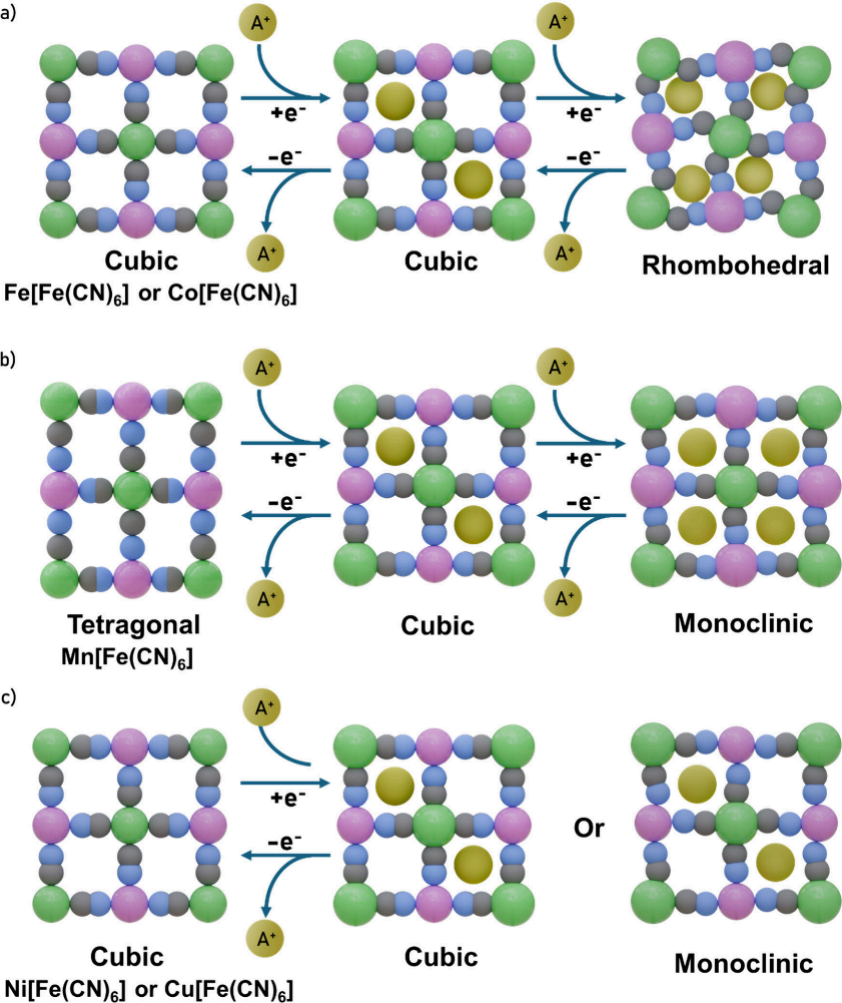
Most common crystallographic phase transition resulting from the insertion or deinsertion of two alkali metal ions in the PBA framework. (a) Cubic to rhombohedral; (b) tetragonal to monoclinic; (c) cubic to monoclinc. The color code used is as follows: R-site (green), T-site (pink), C (gray), N (blue), and Alkali (yellow). Adapted with permission from ref 38. Copyright 2025 Elsevier.

#### 2.1.3 [R­(CN)_6_]_*y*_ Vacancies

The predominant type of vacancies in PBAs, denoted as [R­(CN)_6_]_*y*_, arises from missing hexacyanometallate complexes within the PBA framework.^42^ While [R­(CN)_6_]_*y*_ vacancies fulfill roles akin to counterions in both the composition and crystal structure of PBAs, their electrochemical functions are very distinct.

Composition-wise, missing negatively charged [R­(CN)_6_]^n‑^ units provide an alternative to compensating charges with alkali metals. The formation of [R­(CN)_6_]_*y*_ vacancies is common in most PBAs, including the original PB, but is unfavorable in PBAs that combine trivalent metal cations, as the negative and positive charges are inherently balanced, as in the case of Fe^III^[Fe^III^(CN)_6_].^43^ Thus, PBAs with compositions like T^II^[R^III^(CN)_6_]_1_-_*y*_ or T^III^[R^II^(CN)_6_]_1_-_*y*_, (e.g., Mn^II^[Co^III^(CN)_6_]_0.66_ or Fe^III^[Fe^II^(CN)_6_]_0.75_),^44,45^ do not include a counterion in their formula. Instead, the [R­(CN)_6_]_*y*_ vacancies account for the charge balance, resulting in a neutral compound.

In addition, [R­(CN)_6_]_*y*_ vacancies lead to the coordination of water molecules to unsaturated sites in the metallic centers (see more in the *structural water molecules* subsubsection). Note that, in the literature, the chemical composition of PBAs containing vacancies can be found expressed as whole numbers rather than fractional units (e.g., Fe_4_^III^[Fe^II^(CN)_6_]_3_ instead of Fe^III^[Fe^II^(CN)_6_]_0.75_). This naming difference is due to the lack of a convention in the formulation of these compounds.

Structurally, [R­(CN)_6_]_*y*_ vacancies exert a stabilizing effect in the crystalline structure and can prevent Jahn–Teller distortions. This effect was observed in Mn^III^[Fe^II^(CN)_6_]_0.83_, which displayed a cubic phase with higher vacancy content, compared to Mn^III^[Fe^II^(CN)_6_]_0.93_, which displayed a tetragonal phase with lower vacancy content.^46^ Moreover, the presence of vacancies interconnects several small pores and leads to the formation of larger pores (Figure 4a). In such cases, vacancies can be used for accommodating extra counterions in a cubic lattice while minimizing structural distortions.^9^ For instance, this is the case for K_1.56_Ni^II^[Fe^II^(CN)_6_]_0.91_ in which the presence of vacancies allows hosting more than one potassium per formula unit without giving rise to important distortions in the cubic phase.^47^

**Figure 4:**
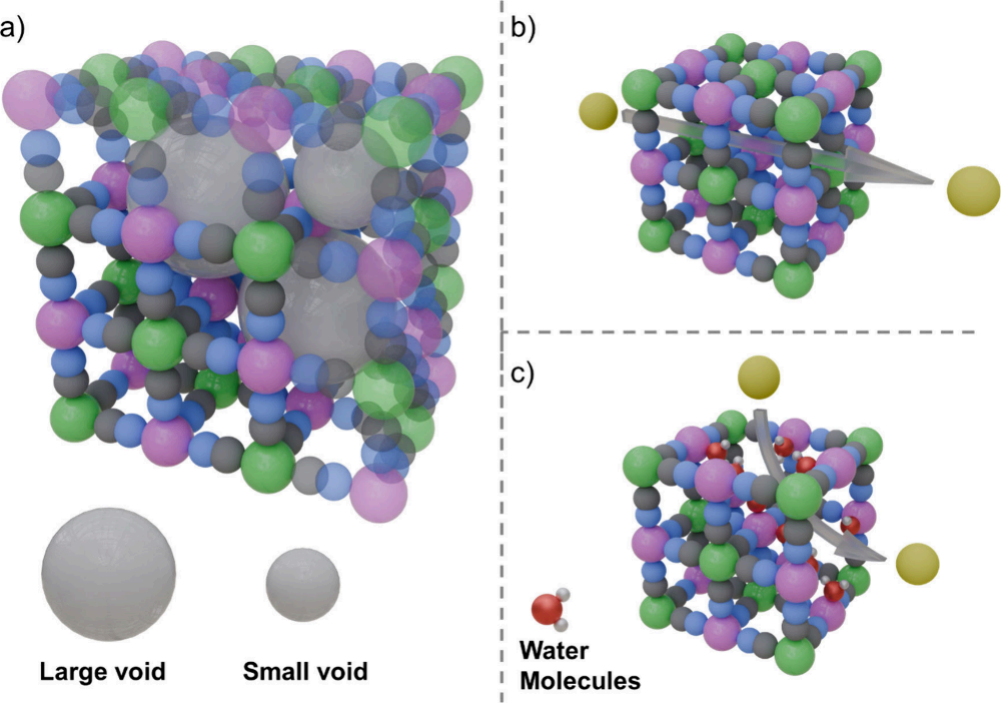
(a) Expanded PBA framework (faded) with [R­(CN)_6_]_*y*_ vacancies (water molecules omitted) displaying two types of voids due to the interconnection of pores; (b) flow of alkali ions across the ⟨100⟩ channels in a vacancy free framework; (c) Improved ion mobility in the framework due to the presence of [R­(CN)_6_]_*y*_ vacancies. The color code used is as follows: R-site (green), T-site (pink), C (gray), N (blue), O (red), H (white), and Alkali (yellow).

Additionally, owing to the same principle, the presence of [R­(CN)_6_]_*y*_ vacancies can have a stabilizing effect on the structure during electrochemical insertion or deinsertion of alkali metals, thereby enhancing cyclability.^46^ Nonetheless, while the introduction of vacancies seems to have positive structural implications, a maximum number of [R­(CN)_6_]_*y*_ vacancies (theoretically 33%) can be introduced before the material becomes structurally unstable as a result of a lack of [R­(CN)_6_]^n‑^ supports.^45^

Electrochemically, [R­(CN)_6_]_*y*_ vacancies play three major roles. First, an increase in vacancy concentration reduces the number of active centers, leading to lower capacities.^48^ Second, these vacancies facilitate the diffusion of alkali metals within the PBA framework, enhancing bulk ionic conductivity and providing alternative pathways for ion transport (Figure 4b and c).^49^ Third, while PBAs inherently exhibit low electronic conductivity (generally between 10^–3^–10^–9^ S cm^–1^),^50–52^ this conductivity further declines with increasing vacancy content, hindering electrochemical ion storage kinetics.^50^ Consequently, researchers have focused on finely tuning vacancy content to strike an optimal balance between cyclability, capacity, ion insertion kinetics, and transport, ultimately improving battery performance^53–56^

#### 2.1.4 Water Molecules

Two distinct types of structural water molecules have been identified in PBAs using neutron and X-ray diffraction (XRD) on single-crystal and powder samples: zeolitic (or interstitial) water and coordinated water.^57,58^ Zeolitic water occupies the voids within the cavities formed by the T­[R­(CN)_6_]^n‑^ complexes and can be easily removed by heating the material below 200 °C.^59^ In contrast, coordinated water is closely associated with the presence of [R­(CN)_6_]_*y*_ vacancies, playing a crucial compositional and structural role by completing the coordination environment of transition metals with uncoordinated sites created by vacancies (Figure 4c). Consequently, removing coordinated water molecules from T-site cations requires heating the PBA to temperatures up to 300 °C due to the strong nature of their bonds.^60^

Electrochemically, zeolitic water can adversely affect PBAs’ conductivity^51^ and performance by competing with alkali metals for space in the PBA pores.^9^ Moreover, zeolitic water tends to solvate alkali ions, reducing ion diffusion within the framework due to the increased volume of the solvated alkali metal.^55^ Similarly, water coordinated to transition metals can impede the movement of alkali metals through the PBA framework via steric hindrance, further diminishing ion diffusion.^60^ Thus, reducing water content in PBA electrodes has been established as a promising route to enhance electrochemical performance.^59^

### 2.2 Role of Active and Inactive Metals on PBA Electrochemical Properties

All the peculiarities described earlier, associated with composition and structure, play a major role in shaping the final electrochemical processes of the PBA. However, the choice of both R-site and T-site cations is the critical factor influencing these processes, with each contributing in distinct yet interconnected ways. To understand their impact, it is essential to examine their individual roles.

Starting with the R-site cation, its chemistry ultimately defines the cathodic or anodic nature of the PBAs. Beginning from higher oxidation states, the redox couple associated with the R-site cation (the metal linked to the C) typically follows a pattern: the higher the atomic number of the R-site cation, the higher the redox potential.^19^ For instance, Fe^III/II^-C_6_ redox couples are typically centered at positive electrochemical potentials from 0.7 to 1.1 V vs SHE (Scheme 1). Conversely, Mn^III/II^-C_6_ or Cr^III/II^-C_6_ redox couples are centered at more negative redox potentials ranging from 0.3 to −0.1 V and −0.5 to −0.9 V vs SHE, respectively (Scheme 1).^19^ At lower oxidation states, the potential for the redox couples is always centered at lower values compared to higher oxidation states. Thus, for example, the Mn^II/I^-C_6_ redox couples are centered at potentials ranging from −0.4 to −0.7 V vs SHE.^19^

**Scheme 1:**
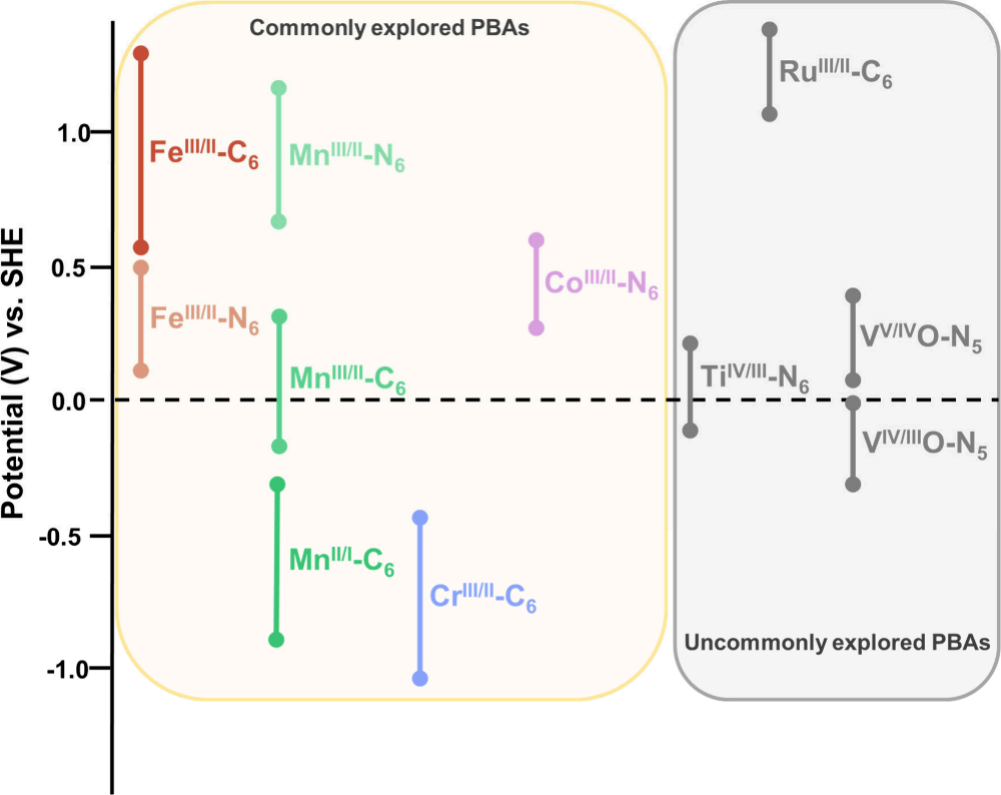
Redox Potentials for the Most Common R^III/II^-C_6_, R^II/I^-C_6_, and T^III/II^-N_6_ Redox Couples^a^

a Commonly explored PBAs’ redox potentials are shown in bright colors, while the uncommonly explored PBA redox pairs are in gray.^58,62^ .

Across the first transition metal row of the periodic table, V, Cr, Mn, Fe and Co, are suitable elements to occupy the R-site position, as they give rise to R­(CN)_6_ coordination complexes in PBAs.^22,61^ Notably, PBAs incorporating V at the R-site are prone to oxidation and have primarily been explored in the field of molecular magnetism,^61,62^ while their electrochemical properties remain largely unexplored to the best of our knowledge. In contrast to the rest of the first row of transition metals, Ni and Cu typically favor square planar R­(CN)_4_ coordination, preventing the formation of the characteristic cubic PBA structure when combined with T-site metals. Beyond the first-row transition metals, Ru and Os can also form cubic PBAs with six-coordinate R­(CN)_6_ complexes.^63,64^ However, due to the limited natural abundance and high cost of these elements, only Ru has been explored — albeit to a limited extent — as a redox-active material in batteries.^64^ Consequently, the selection of R-site cations for battery applications is largely restricted to Cr, Mn, Fe, and Co, balancing considerations of cost, availability, and stability.

While the R-site defines the primary redox behavior, the T-site cation (the metal connected to the N) also plays a crucial role, either directly participating in redox processes or modulating the redox potential of the R-site. For some metals, the T-site can act as an additional active site, allowing an increase in the number of redox processes, as seen in compounds like Na_1.28_Fe^II^[Fe^II^(CN)_6_]_0.88_ or Na_1.58_Mn^II^[Fe^II^(CN)_6_]_0.80_.^30^ Alternatively, the T-site metal fine-tunes the redox potential of the R-site cation, regardless of its own redox activity.^9^ In this case, the following trend applies: the higher the ionic potential of the T-site cation (the ratio of charge to radius), the higher the redox potential of the R-site cation. For instance, in the series Fe^II^[Cr^III^(CN)_6_], Mn^II^[Cr^III^(CN)_6_], Cr^II^[Cr^III^(CN)_6_], the measured redox potentials for the Cr^III/II^-C_6_ reduction process are −0.86, −0.80, and −0.56 V vs SHE, respectively.^65^ Therefore, changing the T-site cation allows for tuning the redox potential for the reduction of Cr^III^ to Cr^II^.

The number of atomic elements suitable for occupying the T-site position is larger compared to R-site cations. Nearly every first-row transition metal can adopt the T-N_6_ coordination environment and has been explored for PBA synthesis, including Ti.^65–67^ More unconventional elements such as Ag^+^, Ga^3+^, and In^3+^ have also been used to prepare PBAs and subjected to electrochemical testing.^65^ Additionally, lanthanides have been shown to form PBAs, though their applications have primarily focused on magnetic properties rather than electrochemical ones.^68^ However, despite this wider selection of available T-site cations, the practical use of more expensive and less abundant metals, like In, Ag, or even lanthanides, remains unfeasible. As a result, most battery materials reported to date based on PBAs rely on first-row transition metals for the T-site position.

With these roles established, the choice of R- and T-site metals becomes pivotal in battery design. The R-site cation, often the redox-active center, determines whether a PBA can function as a cathode or anode material, depending on the position of its redox potential — and of course, on the material choice for the other electrode. Meanwhile, the T-site cation can either participate in redox processes, modulate the redox potential of the R-site, or both. From the point of view of battery design, PBAs with higher atomic number R-site transition metals — from d^6^ electronic configurations onward — are typically employed as cathode materials in aqueous Na- and K-ion batteries due to their electrochemical processes occurring at high redox potentials. These are generally Fe-based or Co-based PBAs, with Fe and Co referring to the active R-site metals. Conversely, PBAs with lower atomic number R-site transition metals — from d^5^ electronic configurations backward — are usually used as anode materials, as their redox processes take place at lower redox potentials. These include Mn-based and Cr-based PBAs, though Mn-based PBAs can also serve as cathodes due to their intermediate redox potentials.^39,69^

### 2.3 Solution-Based Synthetic Methods

The classic mechanical grinding of ferrocyanide using a mortar and pestle is still being one of the most commonly used synthetic methods for preparing PB.^70,71^ However, such preparation technique presents complications related to control over particle composition, morphology and size dispersity.^72^ As a result, most homometallic and heterometallic PBAs are typically prepared using solution-based synthesis.^73^

There are three main solution-based approaches for the synthesis of PBAs: (i) the decomposition of hexacyanometallate salts, (ii) the coprecipitation of solid PBAs upon mixing hexacyanometallate and transition metal salt solutions (namely Route 1), and (iii) the precipitation of PBAs upon mixing transition metal salts with cyanide salt solutions (namely Route 2).^73^ Given that the decomposition of hexacyanometallates is specific to hexacyanoferrate and hexacyanocobaltate salts, yielding PBAs with a single composition,^74,75^ it will not be covered here.

Route 1 entails mixing an aqueous solution of sodium or potassium hexacyanometallate salt, A_4_R^II^(CN)_6_ or A_3_R^III^(CN)_6_ (A = Na^+^, K^+^; R = Cr, Mn, Fe, or Co), with an aqueous solution containing divalent or trivalent cations, mostly transition metal cations, T^II/III^ (T = Cr, Mn, Fe, Co, Ni, Cu, Zn, etc.).^4,19,76,77^ Mixing hexacyanometallate (II) or (III) salts with divalent or trivalent transition metal salts results in the precipitation of PBAs with the chemical formula A_*x*_T­[R­(CN)_6_]_1–*y*_·wH_2_O. Here, “x”, “y”, and “w” represent the number of alkali ions, hexacyanometallate vacancies, and zeolitic water molecules, respectively. These parameters are all interrelated and will vary to achieve a neutral coordination compound.

Four possible combinations can yield PBAs depending on the different oxidation states of the transition metal salts and the hexacyanometallate complexes, as shown by the equations:

1 $$A_{4}R^{\mathrm{II}}{\left(\mathrm{CN}\right)}_{6}+{T^{\mathrm{II}}\mathrm{Cl}}_{2}\to A_{x}{T^{\mathrm{II}}\left[R^{\mathrm{II}}{\left(\mathrm{CN}\right)}_{6}\right]}_{1-y}\cdot {\mathrm{wH}}_{2}O+\mathrm{ACl}$$

2 $$A_{3}R^{\mathrm{III}}{\left(\mathrm{CN}\right)}_{6}+{T^{\mathrm{II}}\mathrm{Cl}}_{2}\to {A_{x}T^{\mathrm{II}}\left[R^{\mathrm{III}}{\left(\mathrm{CN}\right)}_{6}\right]}_{1-y}\cdot {\mathrm{wH}}_{2}O+\mathrm{ACl}$$

3 $$A_{4}R^{\mathrm{II}}{\left(\mathrm{CN}\right)}_{6}+{T^{\mathrm{III}}\mathrm{Cl}}_{3}\to A_{x}{T^{\mathrm{III}}\left[R^{\mathrm{II}}{\left(\mathrm{CN}\right)}_{6}\right]}_{1-y}\cdot {\mathrm{wH}}_{2}O+\mathrm{ACl}$$

4 $$A_{3}R^{\mathrm{III}}{\left(\mathrm{CN}\right)}_{6}+{T^{\mathrm{III}}\mathrm{Cl}}_{3}\to {T^{\mathrm{III}}\left[R^{\mathrm{III}}{\left(\mathrm{CN}\right)}_{6}\right]}_{1-y}\cdot {\mathrm{wH}}_{2}O+\mathrm{ACl}$$

Thus, the choice of hexacyanometallate and transition metal salts will significantly influence the chemical composition of the final PBA.^19^ For example, combining divalent transition metal cations with hexacyanometallate (II) salts (eq 1) will usually give rise to PBAs with more than one alkali metal in the chemical formula (e.g., K_1.56_Ni^II^[Fe^II^(CN)_6_]_0.91_; *x* > 1, y = 0.09).^47^ On the contrary, if the transition metal cations from the hexacyanometallate complex and the transition metal salt precursors are trivalent eq 4, the chemical formula will generally contain no alkali metals (e.g., Fe^III^[Fe^III^(CN)_6_]; x = 0, y = 0).^78^ These differences in the chemical composition have a major impact on the crystal structure of the PBA, leading to different crystallographic phases (as discussed in detail in *Composition and Structural Features of PBAs*).

Route 2 involves mixing an aqueous solution containing a divalent transition metal T^II^ (T = Mn, Co) with an aqueous solution containing an excess of alkali cyanide salt ACN (A: Na^+^, K^+^).^19^ This route yields homometallic PBAs with chemical composition A_*x*_T­[T (CN)_6_]_1–*y*_·wH_2_O, as represented by the equation:

5 $$6\mathrm{ACN}+{T^{\mathrm{II}}\mathrm{Cl}}_{2}\to A_{x}{T^{\mathrm{II}}\left[T^{\mathrm{II}}{\left(\mathrm{CN}\right)}_{6}\right]}_{1-y}\cdot {\mathrm{wH}}_{2}O+\mathrm{ACl}+\mathrm{ACN}$$

Route 2 shares several similarities with the first case in Route 1, and these homometallic PBAs often incorporate two alkali metals in their chemical formula (e.g., Na_1.96_Mn^II^[Mn^II^(CN)_6_]_0.99_; x = 2).^39^

### 2.4 Strategies for Synthetic Control over PBAs Properties

While the described synthetic routes offer an overview of obtaining PBAs, chemists have gained significant control over the final physicochemical properties of PBAs by tailoring the synthetic conditions. These modifications can include adjusting the pH levels of the reaction media, adding an excess of alkali salts to the reaction, or complexing metallic salts among others.^73,79^

To control the composition of PBAs, complexing agents such as citrate molecules have been added during the synthesis, slowing down the formation kinetics of the T­[R­(CN)_6_] complexes, which results in the prevention of vacancy formation. In Na_1.85_Co^II^[Fe^II^(CN)_6_]_0.99_, suppressing vacancies leads to improved material performance in terms of capacity and cyclability when used as a cathode in a battery.^56^ Alternatively, the composition of the PBAs can be controlled by the presence of excess alkali salt during the PBA synthesis. The electrostatic attraction between the alkali metals (A^+^) and the hexacyanometallate complexes ([R­(CN)_6_]^n‑^) gives rise to a templating effect that significantly prevents the formation of vacancies in the material.^53,80^ This strategy has been exploited during the synthesis of Na_*x*_Mn^II^[Fe^II^(CN)_6_], where increasing amounts of alkali metals during the synthesis lead to PBAs with higher alkali metal content and fewer vacancies in the final composition.^53^

For controlling particle characteristics such as shape, surface composition, and porosity, low pH values enable the partial dissolution of the metals during and/or after the formation of the PBA.^81^ This allows for obtaining of PBAs displaying particular facets or the creation of macropores. For instance, using different concentrations of chloroplatinic acid during the preparation of PB, Fe^III^[Fe^II^(CN)_6_]_0.75_, allows for controlling the shape of PB cubes due to the etching of the (100) facet.^81^ Accordingly, Fe^III^[Fe^II^(CN)_6_]_0.75_ with higher Fe^III^ content exposed on the surface displays higher catalytic activities toward the degradation of rhodamine B.

Other particle characteristics like size, zeta-potential, and hydrophilicity can be controlled by the addition of polymers during the reaction. In this case, the polymer acts as a capping agent during the formation of nanoparticles and prevents the aggregation of several PBA units via sterical impediments.^82^ Therefore, increasing the concentration of polymer in the reaction media usually leads to smaller particles.^83^ For instance, increasing the PVP concentration during PB synthesis has enabled obtaining smaller sized particles that display lower magnetic ordering temperatures lower magnetic ordering temperatures compared to larger PB compared to larger PB particles.

All in all, this ability to fine-tune material properties highlights the chemical versatility of PBAs, allowing them to be adapted for a wide range of specific applications, from simple uses like pigments^84^ to more advanced roles in sensing,^85^ biomedicine,^86^ catalysis,^87^ and energy storage.^20^ As it has been emphasized in the introduction and the previous subsections, it is in the latter domain where PBAs have emerged as highly attractive battery materials. This is primarily due to their open framework structure, which facilitates efficient ion intercalation, and the ability to finely tune their composition to achieve specific redox potentials. As a result, PBAs covering a wide range of electrochemical potentials (high or low) can be designed and explored as both cathode and anode materials.

However, research on PBAs has been uneven, with PBAs displaying high redox potentials (above 0 V vs SHE) — primarily used as cathodes — receiving significantly more attention than PBAs with low redox potentials (below 0 V vs SHE), which serve as anode materials and remain comparatively underexplored. For example, Fe-based PBAs have undergone significant development as cathode materials for intercalation-based aqueous Na- and K-ion batteries, with a well-established understanding of their chemistry, structure, and electrochemical properties, reflected in numerous publications each year.^88^ In contrast, the understanding of feasible aqueous Na- or K-ion battery anode materials, such as Mn- and Cr-based PBAs, remains limited. Among them, Na_1.96_Mn^II^[Mn^II^(CN)_6_]_0.99_ is the most studied, but it still lags far behind Fe-based PBAs in terms of research depth and practical applicability.

Moreover, the scarcely published research on Mn- or Cr-based PBAs frequently lacks critical scientific data, such as the reaction conditions for material preparation or the number of cycles for electrochemical characterization. This deficiency is particularly pronounced in studies conducted more than two decades ago. The absence of such comprehensive data renders this field somewhat opaque for those seeking to delve into it.

The following section aims to highlight the numerous open challenges and questions in the realm of PBAs as anode materials. Therefore, the focus will be placed on the limited number of PBAs exhibiting low redox potentials (PBA anode materials from now on) reported to date, delving into vital difficulties like synthetic methodologies for material preparation and control over material composition. Readers interested in learning more about PBAs as cathode materials (PBA cathode materials) are encouraged to refer to comprehensive reviews on their synthesis^73^ and application as cathodes in different types of batteries,^40,89^ as these PBAs will not be extensively covered in this perspective.

## 3 Challenges

As of today, there are three known combinations of metal cations giving rise to Mn-based PBA anode materials (Zn^II^[Mn^III^(CN)_6_], Mn^II^[Mn^II^(CN)_6_], Cr^II^[Mn^III^(CN)_6_], and four combinations giving rise to Cr-based PBA anode materials (Co^II^[Cr^III^(CN)_6_], Fe^II^[Cr^III^(CN)_6_], Mn^II^[Cr^III^(CN)_6_], Cr^II^[Cr^III^(CN)_6_]).^19^ These correspond exclusively to materials whose electrochemistry has been tested, while others — such as Ni^II^[Cr^III^(CN)_6_], used in molecular magnetism^90^ — have yet to undergo electrochemical characterization. This limited number of metallic combinations stands in stark contrast to the wealth of research on Fe-based PBAs, highlighting a disparity in the development of PBA cathode and anode materials that can be attributed to the distinct challenges encountered at each stage of their preparation, characterization, and electrochemical testing.

Prior to the reaction, the cost, availability, and stability of precursor salts emerge as critical limiting factors for both laboratory and industrial-scale preparation of PBA anode materials. During the reaction, constraints associated with precursor stability in aqueous and acid media impede tuning reaction parameters such as precursors concentration or pH, limiting the preparation of materials with specific compositions or particle properties. Following the reaction, the need for costly characterization techniques to conduct thorough studies of PBAs may hinder a comprehensive understanding of their properties, such as composition or crystal structure. Lastly, during electrochemical testing of PBAs, further limitations arise concerning material inherent properties and stability before and during cycling. Moreover, at this point, the use of costly synchrotron, in situ or operando characterization techniques for comprehension of the material changes occurring throughout cycling is usually necessary.

Furthermore, in many cases, the primary difficulty at each stage is the limited amount of reliable scientific data that can be utilized for verifying the physicochemical properties of the resulting PBA. In this matter, the homometallic Mn^II^[Mn^II^(CN)_6_] PBA is an exceptional case. Reports on this material are usually very complete, including most of the crucial information, which is probably the reason behind its status as one of the most used and studied PBA anode materials today.^4,39,69,91^

Given that a thorough comprehension of the challenges and limitations surrounding the study of PBA anode materials is crucial for advancing the field, an exhaustive examination of each constraint is discussed in the following subsections.

### 3.1 PBA Precursor Salts

PBA anode materials are mostly prepared following Route 1 (see Solution-Based Synthetic Methods subsection) synthesis methodologies starting from hexacyanochromate (III) (Na_3_Cr^III^(CN)_6_ or K_3_Cr^III^(CN)_6_) or hexacyanomanaganate (III) (K_3_Mn^III^(CN)_6_) salts. While these precursors are commercially available in gram-scale quantities, their decreasing availability and high price (around 100 €/g)^92,93^ pose significant barriers for large-scale PBA preparation. Although the synthesis of hexacyanoferrate (III) salts is not easier, as it involves using lethal hydrogen cyanide, their wide commercial availability and affordable price (1 €/g) minimize the difficulty of obtaining them.^94^

If commercial sources are unavailable, precursor salts can be synthesized in the laboratory on a multigram scale. However, existing methodologies are based on outdated reports, and synthesis conditions require strict oxygen-free conditions as they usually involve the preparation of intermediate Cr^II^ or Mn^II^ complexes which are chemically unstable in air.^95–97^ Additionally, confirming the purity of the obtained salts is challenging due to limited data on their characterization, even in the same reports that detail the preparation of the compounds. Thus, the purity of these Cr or Mn precursor salts is often uncertain.

Once the challenges of obtaining the hexacyanometallate (III) salts are overcome, the next hurdle relates to their stability in water. Na_3_Cr^III^(CN)_6_ and K_3_Cr^III^(CN)_6_ salts generally exhibit good chemical stability in water as long as they are shielded from intense light sources and can be handled under ambient air conditions.^96^ However, K_3_Mn^III^(CN)_6_ salts rapidly hydrolyze to MnO_*x*_ or Mn­(OH)_*x*_ in aqueous solutions at neutral pH^98^ or undergo disproportionation to Mn^II^ and Mn^IV^ species in acidic media,^99^ rendering them unstable in aqueous environments.

For this reason, solutions have been proposed to overcome this limitation with the Mn-based precursor. For instance, using aqueous solutions containing sodium or potassium cyanide for dissolving K_3_Mn^III^(CN)_6_ was proposed as the presence of excess cyanide groups stabilizes the Mn^III^ complex, preventing its hydrolysis.^99,100^ Unfortunately, this strategy has limited usability due to the high reactivity between some transition metals and cyanide groups, which yields homometallic PBAs, as shown in Route 2 (see Solution-Based Synthetic Methods). Thus, in some cases, the reaction between aqueous solutions containing potassium K_3_Mn^III^(CN)_6_, an excess of CN^–^, and a transition metal T^II^, will yield two different PBAs, as in the equation:

6 $$K_{3}M^{\mathrm{III}}{\left(\mathrm{CN}\right)}_{6}+\mathrm{KCN}+{T^{\mathrm{II}}\mathrm{Cl}}_{2}\to K_{x}T^{\mathrm{II}}\left[{\mathrm{Mn}}^{\mathrm{III}}{\left(\mathrm{CN}\right)}_{6}\right]+K_{x}T^{\mathrm{II}}\left[T^{\mathrm{II}}{\left(\mathrm{CN}\right)}_{6}\right]+\mathrm{ACl}$$

Alternatively, Pasta and co-workers proposed the addition of the K_3_Mn^III^(CN)_6_ salt in solid state to an aqueous solution containing an excess of the transition metal salt (e.g., T­(NO_3_)_2_ or TCl_2_).^4^ With this approach, the formation of Mn_2_O_3_ is avoided, as the [Mn^III^(CN)_6_]^3–^ units react with the transition metal as soon as they get dissolved in a kinetically favored process over Mn_2_O_3_ formation.

### 3.2 PBA Synthesis

The instability of the K_3_Mn^III^(CN)_6_ in aqueous solutions presents significant challenges in achieving synthetic control over the composition, crystal structure, and particle properties, such as size and shape. Composition-wise, strategies like utilizing citrate complexing agents or excess alkali salts during PBA formation have shown promise in obtaining materials with low vacancy content, as observed in Na_1.85_Co^II^[Fe^II^(CN)_6_]_0.99_ or Na_1.81_Fe^II^[Fe^II^(CN)_6_]_0.83_.^48,56,101^ However, these methods are ineffective for synthesizing heterometallic Mn-based PBAs due to the rapid hydrolysis of K_3_Mn^III^(CN)_6_ in water.^98^ Structural control is also hindered, as PBA structures are typically determined by their composition.^43^ Therefore, any lack of control over composition inevitably translates into poor control over structure. To control particle properties such as size or shape, manipulating pH levels during the reaction has been demonstrated to be effective in PBA cathode materials.^102^ However, when dealing with K_3_Mn^III^(CN)_6_, low pH levels induce precursor disproportionation into Mn^II^ and Mn^IV^ species, thus thwarting this method of control.^99^

Currently, only one known coprecipitation approach based on Route 1 exists for synthesizing heterometallic Mn-based PBAs. It involves dissolving K_3_Mn^III^(CN)_6_ in a 10 mM KCN aqueous solution and mixing it with an aqueous solution containing CrCl_2_. This results in the precipitation of the K_*x*_Cr^II^[Mn^III^(CN)_6_] PBA (K content unknown).^4^ It should be noted that this synthetic approach is only applicable when the T-site cation cannot react with the excess CN^–^ to give an homometallic PBA; otherwise, two PBAs are obtained, as explained earlier (eq 6). However, little information about the characterization of this material is reported beyond its poor crystallinity and low capacity, underscoring a significant lack of control over its properties. The other known heterometallic PBA anode materials, Na_*x*_Zn^II^[Mn^III^(CN)_6_] (Na content unknown),^19^ reportedly follows a similar synthetic approach to that used for K_*x*_Cr^II^[Mn^III^(CN)_6_]. However, this PBA was isolated by a company, and due to patents and bylaws, its synthesis and characterization remain unknown.

For obtaining homometallic Mn-based PBAs in an approach similar to route 1, K_3_Mn^III^(CN)_6_ can be added in solid state to an aqueous solution containing an excess of Mn­(NO_3_)_2_, yielding K_0.11_Mn^II^[Mn^III^(CN)_6_]_0.83_.^4^ However, owing to the addition of the hexacyanometallate salt in solid state, this approach does not allow controlling the particle shape of the final material; neither is known whether control over the composition can be achieved.

Obtaining homometallic Mn^II^[Mn^II^(CN)_6_] via Route 2 seems a better strategy.^103^ This PBA anode material, which has also been used as cathode material, has been very well studied, and control over the composition and particle size and shape has been achieved using different cyanide and metallic salts in solution. For instance, under high concentrated conditions, using NaCN for preparing the PBA via Route 2 yields polydisperse cubic particles with a nominal composition of Na_1.96_Mn^II^[Mn^II^(CN)_6_]_0.99_·2H_2_O.^39^ However, under similar synthetic conditions, using KCN during the synthesis yields irregularly shaped K_1.72_Mn^II^[Mn^II^(CN)_6_]_0.93_·3.64H_2_O particles.^103^ Therefore, just by changing the alkali metal of the cyanide salt the particle shape can be controlled. Alternatively, diluted conditions when using NaCN for preparing the material yield a PBA with composition Na_1.24_Mn^II^[Mn^II^(CN)_6_]_0.81_·2.1H_2_O,^104^ which contains a larger number of [Mn­(CN)_6_]_*y*_ vacancies (y = 0.19) compared to the almost vacancy-less Na_1.96_Mn^II^[Mn^II^(CN)_6_]_0.99_·2H_2_O (y = 0.01) obtained under concentrated conditions. Thus, in this case, lowering the concentration of the cyanide salt in the reaction allows for decreasing the vacancy content in the material. Moreover, larger particles can also be obtained in diluted conditions.

The enhanced chemical stability of K_3_Cr^III^(CN)_6_ compared to its Mn counterpart in aqueous solutions offers many possibilities for controlling the composition, structure, and particle properties of the resulting Cr-based PBAs. Strikingly, these possibilities have been scarcely explored for Cr-based PBAs. Starting with heterometallic Cr-based PBAs, Mn^II^[Cr^III^(CN)_6_] is the most studied anode material. Two reports have demonstrated the successful synthesis of this material.^77,105^ In one report, following Route 1, aqueous solutions of K_3_Cr^III^(CN)_6_ and MnCl_2_ are mixed_,_ yielding the material with composition K_0.01_Mn^II^[Cr^III^(CN)_6_]_0.72_.^105^ On the other hand, the second report proposes the use of a mixture of Route 1 and 2 by mixing a NaCN solution with an excess of CrCl_3_ and HCl. After refluxing the solution for a few hours, Mn­(NO_3_)_2_ is added and a precipitate with composition Na_0.04_Mn^II^[Cr^III^(CN)_6_]_0.70_ is obtained.^77^ In both cases, the PBAs present a large number of [Cr^III^(CN)_6_]_*y*_ vacancies (y ∼ 0.3), and therefore small amounts of alkali metal are hosted in the framework. Despite using very different methodologies, both materials have very similar core metal compositions. However, using NaCN in the second example seems to allow forcing the introduction of Na in the final material, and slightly tune the number of vacancies. Moreover, the methodology presented in the latter case seems to allow for partial control over particle size and shape as demonstrated by the low particle size distribution achieved in the final cubic microparticles. Nonetheless, additional tuning of the synthetic conditions is needed to determine whether the particle properties can be further tuned.

Interestingly, the field of molecular magnetism has made strides in the control of composition and material properties of Cr-based PBAs. For instance, very good control over Fe^II^[Cr^III^(CN)_6_] particle composition and size has been reported using Route 1 approaches while taking advantage of emulsions.^106^ Thus, cubic nanocrystals with sizes ranging from 2 to 50 nm can be obtained by using IGEPAL CO-520 as an emulsifier when mixing K_3_Cr^III^(CN)_6_ with FeCl_2_ in cyclohexane. Moreover, adding different amounts of KCl to the reaction allows to finely tune the number of [Cr^III^(CN)_6_]_*y*_ vacancies in the system, yielding materials with composition K_0.05_Fe^II^[Cr^III^(CN)_6_]_0.68_, K_0.2_Fe^II^[Cr^III^(CN)_6_]_0.73,_ K_0.35_Fe^II^[Cr^III^(CN)_6_]_0.78_ upon increasing the amount of KCl added to the reaction. Strikingly, their electrochemical characterization has not been reported to date.

Alternatively, instead of using coprecipitation approaches, KCo^II^[Cr^III^(CN)_6_], KNi^II^[Cr^III^(CN)_6_], and KFe^II^[Cr^III^(CN)_6_] have been prepared layer-by-layer using thin film Langmuir Blodget approaches,^107^ demonstrating the possibility to control the orientation growth of the PBAs. In the prepared films, the particles show a preferential growth along the (h, 0, 0) plane due to the use of octadecylamine (ODA) as a surfactant. However, in this case, the details of the composition of the materials are not reported.

Beyond heterometallic Cr-based PBAs, homometallic K_0.01_Cr^II^[Cr^III^(CN)_6_]_0.67_ has been recently prepared using Route 1 with a small modification.^108^ In this case, an aqueous solution of CrCl_2_ is added very slowly (10 mL h^–1^) to an aqueous solution of K_3_Cr­(CN)_6._ Standard characterization of the material’s morphology reveals that the very slow addition of the metallic salt allows for control over the particle size and shape, yielding 20–70 nm nanocubes. However, further tuning of the reaction conditions needs to be carried out to achieve a rationalization between the particle composition and synthetic conditions.

Thus, control over some particle characteristics (mostly size and shape) has been demonstrated only in a few reports, taking advantage of compelling strategies like using complexing agents or polymers during material synthesis to control the number of vacancies or tune particle size. These synthetic strategies represent promising approaches to advance the preparation of PBA anode materials with tunable particle characteristics. Obtaining such control is pivotal to overcoming important concerns about reproducibility, a subject under interrogation in some metal–organic framework systems lately.^109,110^

### 3.3 PBA Characterization

Once synthesized, thorough characterization becomes imperative to comprehend the composition, crystal structure, and particle properties of PBA anode materials. This knowledge is essential for correlating the material’s physicochemical and electrochemical properties.^20^ However, achieving comprehensive characterization for PBAs can be costly and complex.

For composition analysis, energy dispersive X-ray measurements (EDX) offer a cost-effective method for qualitatively estimating the metallic composition of PBAs, striking a balance between cost and time.^111^ However, proper characterization of PBA composition requires inductive coupled plasma-mass spectroscopy (ICP-MS), enabling quantitative analysis of core metals, and thus allowing an estimation of the number of vacancies.^112^ While ICP is an excellent technique for precisely determining the composition of heavier metals, it is not as effective in characterizing lighter elements, especially in low concentrations.^113^ Additionally, the determination of alkali metals can be problematic due to interferences from other species.^114^ Thus, characterizing vacancy rich materials, which usually have low alkali metal content, can become a difficult task using ICP. Moreover, ICP is a time-consuming technique and can be costly due to the required use of standards. X-ray photoelectron spectroscopy (XPS) is another technique that has become predominantly available in many laboratories over the past decade. XPS can provide detailed quantitative analysis of the atomic surface composition of PBA particles. However, its main limitation lies in the sampling depth — typically 10 nm — though this can vary depending on the X-ray source, as higher-energy sources impart more kinetic energy to electrons, allowing them to escape from deeper within the sample.^115^

Thermogravimetric analysis (TGA) will be necessary to determine the water content in PBAs.^116^ However, characterizing different types of water (zeolitic and coordinated) using TGA can be challenging, depending on the material chemistry. As mentioned earlier, zeolitic water and coordinated water can require up to 200 and 300 °C, respectively, to be removed.^59,60^ Due to the different bonds that water molecules can form with different metals, weakly bound coordinated water might be removed at similar temperatures to those required for removing zeolitic water, thus masking the identification of types of water.^117^ On the other hand, strongly bound coordinated water might require temperatures up to 300 °C to be removed.^60^ However, at this temperature, the organic CN^–^ bridges in the PBA can begin to decompose, possibly masking and preventing the accurate determination of water content.^118^

Structurally, determining the crystallographic phase and phase purity of the PBA requires the use of XRD, typically with a diffractometer equipped with a stage for powder samples.^119^ XRD is valuable for obtaining additional information about the crystallographic features of PBAs, such as preferential facet growth^120^ or the presence of vacancies.^121^ Achieving accurate results necessitates a very low noise-to-signal ratio, which implies prolonged measurements. Difficulty still arises if those features are not very prominent in the material. Moreover, to gain a proper understanding of the crystalline features being analyzed, comparing experimental and predicted patterns will be necessary to reach any conclusion.^122^ Nonetheless, determining the content of vacancies or preferential facet growth in PBAs using only XRD is not conclusive, specially if those features are not very prominent in the material. Fourier transformed infrared spectroscopy (FTIR) can be a very useful technique to confirm the orientation of the cyanide linkages between transition metal cations.^123^ However, the presence of impurities complicates the task of assigning FTIR signals due to the overlapping of vibrational modes.^124^

Particle characteristics, including morphology and size, can be determined through scanning electron microscopy (SEM).^125^ SEM allows for easy recognition of features like preferential facet growth or facet etching, aiding in the characterization process. However, imaging some PBAs can pose a challenge due to the limited conductivity of these materials^126,127^ and particle aggregation in powdered samples, leading to local charging that disrupts imaging.^128^ Ensuring good contact between the sample and the sample holder can avoid this problem. Thus, coating the sample with a conductive material like gold might be a requirement for enhancing imaging quality.^129^ Transmission electron microscopy (TEM) is another valuable technique for characterizing morphology and particle size, especially beneficial for smaller particle sizes.^130^ Moreover, a TEM equipped with additional electron diffraction or EDX detectors can be a powerful instrument for determining the crystal structure and composition of PBAs.^131^ However, there are limitations to analyzing PBAs with TEM, notably due to the instability of the material under the electron beam. PBAs are metal–organic materials and subjecting them to highly accelerated electron beams can result in the decomposition of the cyanide groups and therefore the collapse of the material.^132^ Dynamic light scattering (DLS) can be used as a complementary technique to SEM and TEM for determining the size of nanosized PBAs.^133^ However, most PBA particles used in battery systems are typically large and polydisperse, making the results obtained from DLS meaningless.

More advanced characterization techniques, such as X-ray absorption (XAS) or small-angle X-ray scattering (SAXS) can provide deeper insights into the PBA composition and structure.^134^ However, while benchtop XAS and SAXS instruments have become more available in recent years, these techniques are either really expensive or predominantly found in synchrotron facilities, limiting accessibility.

As it is shown, characterizing and understanding PBA anode materials is, in general, a difficult task that can be really costly depending on the number and quality of the techniques used. Moreover, as will be discussed in the next subsection, having great characterization does not ensure the good performance of the material, and further investment in in situ or operando techniques is necessary to understand why PBA anode materials are underperforming.

### 3.4 PBAs’ Electrochemistry

After synthesizing the PBA anode materials and thoroughly characterizing their composition and properties, the most challenging phase remains: unveiling their electrochemical activity. Although seemingly straightforward, this step often exposes a critical limitation — many PBA anode materials fail to exhibit electrochemical activity. This stands in stark contrast to PBA cathode materials, which consistently show some degree of electrochemical response, regardless of their ultimate performance.

This lack of activity in PBA anode materials is hypothesized to stem from their intrinsic instability and physical properties. The instability likely arises from two key factors: ligand isomerism and structural degradation during electrochemical cycling. On the other hand, their physical limitations — particularly low conductivity — induce electrode polarization, further impairing performance. Addressing these interconnected challenges is crucial to enhance stability and unlock the full electrochemical capabilities of PBA anode materials.

#### 3.4.1 Cyanide Ligand Isomerism

Cyanide isomerism was first observed in KFe^II^[Cr^III^(CN)_6_] during the 1960s.^135^ The material, initially orange, gradually turned green upon heating. FTIR analysis revealed a significant shift in the CN^–^ stretching frequency, from 2168 to 2092 cm^–1^, following thermal treatment. This frequency shift was linked to a reorientation of the CN^–^ molecule, transitioning from a Cr–C≡N–Fe to Cr–N≡C–Fe (Figure 5).^136^

**Figure 5:**
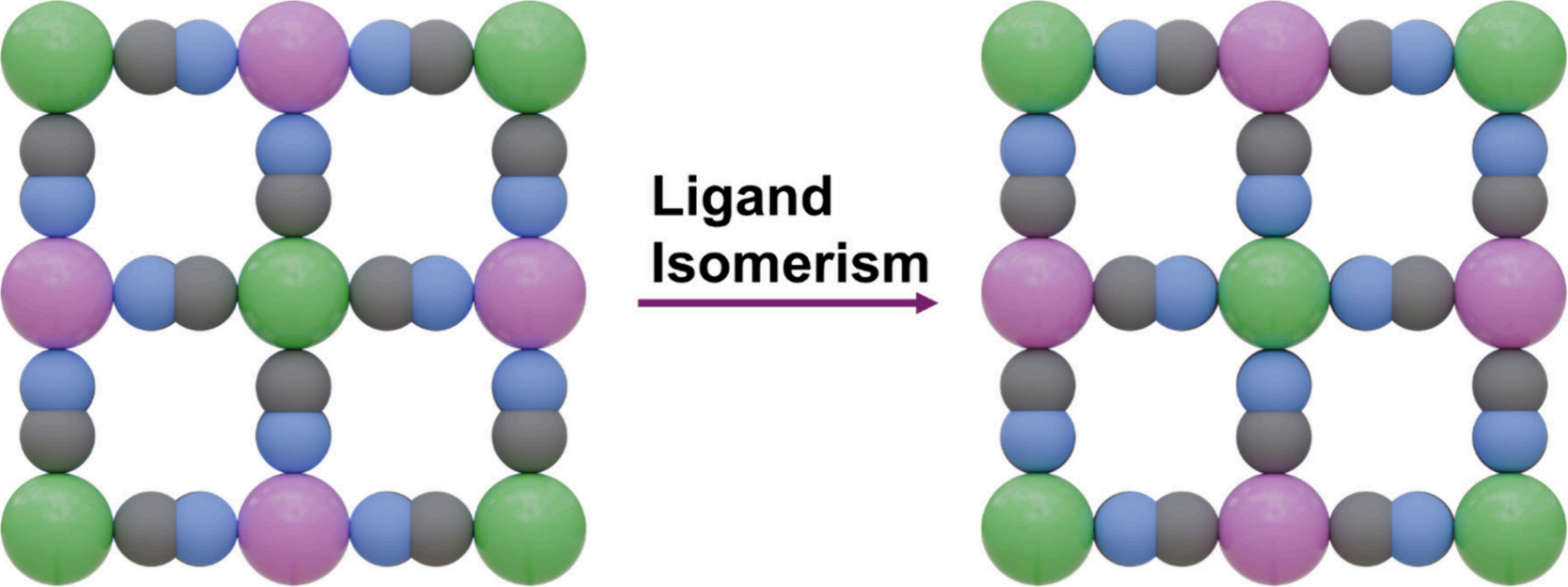
Graphic representation of the cyanide ligand isomerism in PBAs. The color code used is as follows: R-site (green on the left, pink on the right), T-site (pink on the left, green on the right), C (gray), and N (blue).

The occurrence of linkage isomerism in PBAs is primarily governed by the stability of the R-C_6_ bonds. Theoretical and experimental studies have shown that when the T-site cation (N-coordinated) forms a more stable complex in the R position (C-coordinated), linkage isomerism is favored.^135,137^ The stability of the four most commonly used hexacyanometallate units follows this order:

$${\left[\mathrm{Fe}{\left(\mathrm{CN}\right)}_{6}\right]}^{n-}\approx {\left[\mathrm{Co}{\left(\mathrm{CN}\right)}_{6}\right]}^{n-}\gg {\left[\mathrm{Cr}{\left(\mathrm{CN}\right)}_{6}\right]}^{n-}>{\left[\mathrm{Mn}{\left(\mathrm{CN}\right)}_{6}\right]}^{n-}$$

From a synthetic perspective, PBA anode materials with compositions A_*x*_Fe­[Cr­(CN)_6_] or A_*x*_Co­[Mn­(CN)_6_] can be reliably produced. However, depending on the kinetics of the linkage isomerism, the composition may shift to A_*x*_Cr­[Fe­(CN)_6_] or A_*x*_Mn­[Co­(CN)_6_].^138^ which further complicates the design of stable PBA anode materials. This phenomenon limits the number of viable compositions, presenting a significant challenge in the field.

The presence of linkage isomerism significantly impairs the electrochemical performance of PBA anode materials. As discussed earlier, R-C_6_ complexes containing Mn or Cr (which have lower redox potentials) are less stable than those with Fe or Co. Therefore, the transition of Cr or Mn from an active to an inactive position (and vice versa for the Fe or Co) poses significant electrochemical challenges, as this shift directly impacts the material’s redox behavior, shifting the main redox processes from a lower to a higher redox potential. This instability further complicates the use of these materials as anodes.

Despite these challenges, linkage isomerism is a thermodynamically regulated equilibrium process. As a result, complete isomerization is rare and typically occurs only after thermal treatment at elevated temperatures. Importantly, this process can be partially mitigated by carefully controlling the temperature during synthesis, allowing for better stability in the resulting materials.

#### 3.4.2 PBA Instability During Electrochemical Cycling

The degradation of PBAs in aqueous electrolytes during electrochemical cycling has long been a subject of research.^33^ This phenomenon, resulting in the dissolution of the PBA, has been observed since the inception of these materials as battery electrodes and becomes obvious due to the coloration of the aqueous electrolyte. Although the dissolution of PBA electrodes is not exclusive to PBA anode materials, it holds particular significance for them compared to PBA cathode materials due to their lower stability.^77^ Yet, the dissolution of PBA anode materials has been scarcely studied. However, findings derived from studies on cathode materials Na_2_Ni^II^[Fe^II^(CN)_6_] and Na_2_Co^II^[Fe^II^(CN)_6_] using operando ICP in a flow cell have provided valuable insights into dissolution mechanisms.^139^

The conditions under which PBAs undergo electrochemical cycling, such as the pH of the electrolyte or the chemical nature of the anion in the electrolyte salts, have a major influence on the material’s stability.^41,139–141^ In alkaline electrolytes (pH ≈ 12.7), PBAs reportedly dissolve due to the adsorption or attack of hydroxyl ions (OH^–^) to both T- and R-site cations, resulting in precipitated metal oxyhydroxides (TOOH) or oxides (TO_*x*_) and dissolved hexacyanometallate complexes [Fe^III^(CN)_5_OH]^3–^.^41,139^

Under near-neutral pH conditions (pH ≈ 6), typical for battery operation, specific adsorption of electrolyte anions onto the PBA surface drives electrode degradation. The three-step proposed dissolution mechanism at neutral pH is noteworthy: during the electrochemical oxidation of the electrode, anion species (An^–^) from the electrolyte adsorb onto the PBA surface, facilitating the deintercalation of alkali ions, A^+^ (Figure 6a).^41,139,140^ However, since the electrode–electrolyte interfaces are typically charged beyond the potential of zero charge, some anions remain on the surface to compensate for the overcharge, forming metastable surface complexes, particularly at positively charged R- and T-site positions, R–C≡N–T···An*^ads^* and An*^ads^* ···R–C≡N–T.^41,139^ Consequently, significant distortions occur in the R–C≡N–T bond due to the formation of these metastable complexes (Figure 6b). Finally, upon the reduction of the PBA, alkali ions are inserted back into the crystal structure, leading to considerable structural strain. At this point, the distorted R–C≡N–T bond breaks at its weakest point, releasing T^n+^ into the solvent (Figure 6c), while the stronger affinity of the adsorbed anion for the R-site cation gives rise to insoluble R­(CN)_*x*_An_*z*_ complexes.^139^

**Figure 6:**
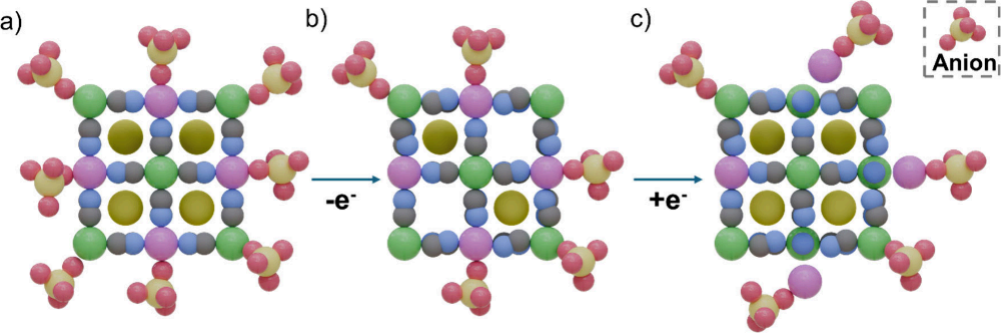
Three-step mechanism of PBA dissolution: (a) anion adsorption to R- and T-site cations facilitating alkali metal deintercalation; (b) some anions remain on the surface due to overcharge, distorting the R–C≡N–T bond; (c) dissolution of the T-site cations upon intercalation of alkali metals. The color code used is as follows: R-site (green), T-site (pink), C (gray), N (blue), O (red), S (pale yellow), and Alkali (yellow).

At low pH (pH ≈ 2.3), H_3_O^+^ species intercalate into the PBA structure, leading to the protonation of the nitrogen atoms in the CN- ligands. This protonation weakens the R–C≡N–T bond, ultimately causing the dissolution of both the T-site cation and the [R­(CN)_6_]^n‑^ complexes.^41^

To provide more insight into the role of the anion in the dissolution problem, further investigation was carried out at neutral pH with electrolytes containing different anions, such as Na_2_SO_4_, NaClO_4_, NaNO_3_, and NaCl, revealing the crucial role of anion chemisorption in the dissolution phenomenon.^139^ For instance, in the case of Na_2_Ni^II^[Fe^II^(CN)_6_], the adsorption strength follows the order: SO_4_^2–^ > Cl^–^ > NO_3_^–^ > ClO_4_^–^. Therefore, higher adsorption strength leads to faster metal dissolution during cycling.

Furthermore, it was demonstrated that the use of highly concentrated electrolytes (i.e., >7 M salt concentration) for cycling the materials significantly reduces the dissolution of metal cations from the PBA into the solvent.^142^

While this latter point might seem counterintuitive, as one would expect that more concentrated electrolytes lead to more dissolution, it is important to remember that the dissolution of metal ions occurs due to the metal uptake capability of the solvent in a process facilitated by the formation of anion-metal complexes with the electrolyte. Thus, in highly concentrated aqueous electrolytes, the metal uptake capability of H_2_O is lower compared to less concentrated electrolytes due to the lack of excess water, leading to lower PBA dissolution rates. For instance, Na_2_Ni^II^[Fe^II^(CN)_6_] demonstrated 80% capacity retention after 1,000 cycles when cycled in 0.25 M NaClO_4_. In contrast, when cycled in 4 or 8 M NaClO_4_, the material retained 80% of its capacity after more than 10,000 and 100,000 cycles, respectively.^139^

Additionally, it was revealed that the anions seem to significantly influence the effect of pH on the dissolution of the PBA. For example, 0.25 M NO_3_^–^ at lower pH inhibited the formation of OH^–^ species which, combined with the low chemisorption of NO_3_^–^ to Ni^2+^, resulted in 80% capacity retention after 1,600 cycles, compared to 80% retention after 800 cycles at near-neutral pH. In contrast, cycling in 0.25 M SO_4_^2–^ caused faster degradation of the PBA, with 80% capacity retention after 250 cycles, compared to 450 cycles at near-neutral pH with the same electrolyte concentration.^139^ The mechanism behind this behavior of the SO_4_^2–^ at low pH is unclear, but it aligns with previous results observed in other PBAs.

Finally, it is important to note that the stability of anion-metal complexes varies depending on the specific material composition, given the distinct chemical properties of different transition metals.^139^ For instance, during cycling with 0.05 M Na_2_SO_4_ at near-neutral pH, Na_2_Co^II^[Fe^II^(CN)_6_] demonstrated lower stability compared to Na_2_Ni^II^[Fe^II^(CN)_6_], suggesting stronger binding of SO_4_^2–^ anions to Co than Ni.^139^ In cases where the T-site cation is inactive, such as Ni^2+^ in Na_2_Ni^II^[Fe^II^(CN)_6_], minimal distortion of the octahedral coordination occurs during electrochemical cycling. However, when the T-site cation acts as an active metal, changes in oxidation state during cycling can induce additional distortions, possibly leading to increased dissolution.^33^

Thus, the metal composition of the PBA, along with factors such as pH, electrolyte composition and concentration, must be carefully considered when designing PBAs for enhanced cyclability in aqueous media. Moreover, maintaining the electrolyte pH close to neutral during cycling can enhance electrode lifetime, as even small local pH changes resulting from metal or ligand dissolution can significantly impact further electrode degradation^143^ or trigger side reactions, such as the hydrogen evolution reaction (HER) or oxygen evolution reaction (OER) at high overpotentials.^144,145^

PBA anode materials, in particular, exhibit very high solubilities in water and low-concentration electrolytes.^77^ As a result, cycling them in low-concentration electrolytes is often impractical, as they dissolve within the first electrochemical cycle. To mitigate this, highly concentrated NaClO_4_ (10 to 17M) is typically used as the electrolyte for testing these materials. For example, K_0.01_Cr^II^[Cr^III^(CN)_6_]_0.67_·3.8H_2_O showed 80% capacity retention after 200 cycles in 17 M NaClO_4_.^108^ The use of such electrolytes, often referred to as water-in-salt electrolytes, significantly broadens the applicability and sustainability of PBA electrodes by reducing dissolution rates.

However, the lack of exploration into alternative electrolytes presents a considerable barrier to the practical use of PBAs in real-life battery applications. This issue brings up another critical point: the unresolved matter of ion selectivity in PBA anode materials. While ion selectivity in PBA cathode materials is well-documented, as seen in materials like Na_2_Ni^II^[Fe^II^(CN)_6_] and Na_2_Co^II^[Fe^II^(CN)_6_], the role of ion selectivity in PBA anode materials remains unexplored. Notably, most studies have been limited to NaClO_4_-based electrolytes,^19,77,104,105,108^ leaving the behavior of PBA anode materials in electrolytes containing other anions and cations poorly understood.

As an alternative to aqueous media, nonaqueous electrolytes present a compelling option for the electrochemical cycling of Cr- and Mn-based PBAs. Widely adopted in commercial technologies,^146^ these electrolytes have been shown to significantly mitigate the dissolution of Cr- and Mn-based PBAs, thereby enhancing their cyclability.^77^ However, when shifting to nonaqueous systems, more competitive anode materials, such as HCs, present a significantly more cost-effective and higher-performance alternative to PBAs. Moreover, to fully understand the potential of PBAs in nonaqueous environments, a systematic investigation of electrolyte formulations is needed to elucidate the complex interplay between electrolyte composition, material stability, and electrochemical performance. Finally, when designing a battery for a specific application, its operational environment must be carefully considered, as nonaqueous batteries are susceptible to safety risks, including thermal runaway, flammability, and pressure build-up.^147^

#### 3.4.3 Electrode Polarization

In the previous subsection, the instability of PBAs during electrochemical cycling was thoroughly discussed, primarily focusing on external factors such as electrolyte composition, pH, and concentration. However, the inherent properties of PBAs also play a crucial role in their performance. As highlighted in the *[R­(CN)*_*6*_ *]­y Vacancies and Water Molecules* subsubsections, PBAs exhibit intrinsically low electrical conductivity, with reported values for PBA cathode materials typically ranging from 10^–3^–10^–9^ S cm^–1^.^50–52^ This low conductivity negatively impacts PBAs by inducing electrode polarization,^48,138^ slowing ion insertion kinetics, and ultimately reducing electrode performance.^56,148,149^

For PBA anode materials — particularly Mn- and Cr-based PBAs — electronic conductivity has yet to be reported. Nonetheless, based on our experience working with these materials, their conductivity is likely comparable to that of PBA cathodes (between 10^–3^–10^–9^ S cm^–1^),^50–52^ primarily from an optical band gap perspective. When contrasted with state-of-the-art anode materials for aqueous Na- and K-ion batteries,^13,17^ PBA anode materials appear more conductive than the white, insulating NaTi_2_(PO_4_)_3._ ^150^ and KTi_2_(PO_4_)_3_.^151^ This semiconducting to insulating behavior can contribute to electrode polarization effects, akin to those reported for PBA cathodes,^54,56^ and may influence overall anode performance. Nonetheless, such polarization phenomena are not unique to PBAs — they also affect materials like NaTi_2_(PO_4_)_3_ and can often be mitigated through surface coatings (see *Processing PBA Anode Materials with Coatings*).^16,152^ Moreover, polarization does not appear to be a limiting factor in full-cell configurations involving PBAs.^4^

Overall, PBAs tend to perform best in all-PBA battery systems, where both electrodes are well matched in terms of reaction kinetics and structural stability.^104^ Consequently, the suboptimal performance of some PBA anodes likely arises from a combination of intrinsic material limitations and external factors, underscoring the importance of understanding both in order to enhance their performance in energy storage applications.

#### 3.4.4 Understanding PBA Performance

Identifying the root causes of low PBA performance is an exceptionally challenging task. Unraveling the processes behind this lack of activity often requires advanced characterization techniques — such as synchrotron, in situ, or operando methods — which are not only complex but also have limited accessibility.^134^

Among the most conventionally used techniques, synchrotron X-ray diffraction has been employed to obtain high-resolution powder diffraction patterns of PBAs, enabling the determination of lattice parameters of the crystal structure.^9^ Collecting diffraction patterns for pristine, fully oxidized, and fully reduced materials allows tracking the expansion and compression of the lattice and crystal phase transitions via changes in lattice parameter values. In PBA anode materials, these experiments have been exclusively carried out ex situ on Na_1.96_Mn^II^[Mn^II^(CN)_6_]_0.99_·2H_2_O, K_0.11_ Mn^II^[Mn^III^(CN)_6_]_0.83_·3.64H_2_O, and Na_0.04_Mn^II^[Cr^III^(CN)_6_]_0.70_·2.80H_2_O by M. Pasta and collaborators, shedding important light on the structural evolution of the materials upon electrochemical cycling.^4,39,77^

Synchrotron soft X-ray absorption spectroscopy (sXAS) is an insightful technique for determining oxidation states and coordination environments of metal cations in PBAs. Similar to XRD, XAS can be employed to collect data for pristine, reduced, and oxidized samples. This technique was used ex situ to elucidate the existence of Mn^I^ upon electrochemical reduction of Na_1.24_Mn^II^[Mn^II^(CN)_6_]_0.81_·2.1H_2_O. Mn^I^ is an unstable and elusive oxidation state of manganese, and this was one of the first times it had been isolated due to the stabilizing effect of the cyanide linkers.^104^

Similar to ex situ XAS, electron energy loss spectroscopy (EELS) is a cheaper and more available technique (coupled to a TEM or STEM) that allows for determining oxidation states of metals, providing useful information on the changes occurring in the metal cations upon cycling. EELS has been used to monitor oxidation state changes in K_0.01_Cr_3_[Cr­(CN)_6_]_2_·3.8H_2_O upon cycling.^108^ This information, complemented with an XRD study at different stages of the electrochemical cycling, allows monitoring phase and oxidation changes, and therefore obtaining important information on the stability of the material.

Operando XAS during electrochemical cycling of PBA is an interesting technique for determining the oxidation state of the metals as they are cycled, allowing identification of the active and inactive metals.^153^ Operando XRD and Raman spectroscopy, when coupled together, allow obtaining the correlated local structure, crystal structure, redox activity, and potential profiles of PBAs during the charging and discharging processes.^154^ In situ inductively coupled plasma with a scanning flow cell (ICP-SFC) allows the determination of the dissolution of the metals contained in the PBA upon cycling.^139^ However, these latter operando or in situ techniques and other advanced characterization techniques that have not been highlighted have exclusively been used for studying PBA cathode materials.

In conclusion, the field of PBA anode materials faces several significant challenges that have impeded substantial progress. These challenges range from the inherent instability of precursors used in PBA preparation to the dearth of reliable data and exploration of synthetic methodologies, and ultimately to the observed low performance in final PBAs when utilized in battery assembly and testing. Furthermore, the preparation and investigation of these anode materials incur substantial costs, and their comprehensive understanding often relies on the use of characterization techniques that are not only expensive but also not readily accessible. Collectively, these significant barriers render the field of PBA anode materials opaque, hampering progress and necessitating innovative solutions to facilitate their integration into practical applications.

## 4 Past Horizons

To tackle the challenges in the field of PBA anode materials and drive the technology forward, it could be beneficial to revisit successful strategies employed in the development of PBA cathode materials. Among these ‘past horizons,’ several promising yet unexplored strategies for PBA anode materials include fine-tuning chemical reaction conditions in Cr-based PBAs, conducting operando ICP studies in a flow cell, and implementing coatings on PBA anode materials. These approaches hold promise to address key issues related to the understanding of PBA anode materials and enhance their electrochemical performance, thereby contributing to advancements in battery technologies.

### 4.1 Tuning Chemical Reaction Conditions in Cr-Based PBAs

The chemical tunability of PBAs sets them apart from other materials, such as oxides, commonly used in batteries. This tunability has been extensively explored in the field of Fe-based PBAs, with numerous studies investigating modifications in reaction conditions that result in materials with varied compositions, structures, and particle properties.^88,155^

For example, the addition of citrate complexing agents during the reaction has been demonstrated to slow down the formation kinetics of Fe-based PBAs, leading to reduced vacancies in the final material.^56^ Another strategy involves using highly concentrated alkali metal salt solutions as reaction media, where the alkali metals act as templating agents, resulting in materials with minimal vacancies.^53^ Additionally, in terms of particle properties, the use of PVP during the reaction has been reported to regulate facet growth and allow to tune the hydrophilicity of the PBA surface in Fe-based PBAs.^83^

Despite the success in Fe-based PBAs, these synthetic strategies remain largely underexplored in Cr-based PBAs synthesis. Exploring such approaches and addressing this gap between cathode and PBA anode materials is crucial before venturing into alternative methodologies, as these studies will elucidate the extent of tunability achievable in Cr-based PBA anode materials.

### 4.2 Conducting Operando ICP Studies in a Flow Cell

While operando ICP studies have only recently been employed to unveil the dissolution mechanism in Fe-based PBAs, its application could prove pivotal in the development of PBA anode materials.^41,139^ As previously discussed, this technique enables in situ monitoring of metal dissolution during the electrochemical cycling of electrodes. Notably, although PBA anode materials present higher dissolution rates compared to PBA cathode materials,^77^ such comprehensive studies have not been conducted yet. Instead, most in situ techniques used for characterizing PBA anode materials have focused on monitoring structural changes using diffraction techniques. However, correlating these structural changes with metal dissolution is essential.

Altogether, coupling experimental data with complementary theoretical calculations would provide deeper insight into the actual dissolution mechanisms of PBA anode materials. This knowledge could, in turn, be harnessed to design PBAs with tailored properties by fine-tuning reaction conditions, thereby unlocking their capability for use as electrodes.

### 4.3 Processing PBA Anode Materials with Coatings

Processing materials with coatings has gained significant relevance in battery research across all types of battery components, including anodes, cathodes, and separators.^156,157^ Originally designed to mitigate material corrosion and dissolution by shielding electrodes from direct electrolyte exposure through the creation of a physical barrier,^158^ surface coatings have evolved to serve additional functions, including enhancing the performance or safety of the electrodes.^159,160^ Among the most commonly used materials for coatings, polymer materials such as poly­(3,4-ethylenedioxythiophene) (PEDOT),^161^ polyaniline,^162^ and poly­(azure C)^163^ are particularly favored in energy storage research due to their conductive nature and cost-effectiveness.

Despite presenting several benefits, the coating of PBAs has exclusively been explored in cathode materials, proving to be a fruitful approach for enhancing the chemical stability and cyclability of electrodes.^161,164,165^ Therefore, applying coatings to PBA anode materials could represent a cost-effective strategy for improving their electrochemical properties, solving polarization issues, and facilitating their integration into battery systems.

## 5 Future Horizons

Beyond the past horizons, recent advancements in synthetic and characterization techniques, predominantly utilized in PBA cathode materials, have broadened the horizon landscape for developing PBA anode materials suitable for real-world battery systems. Among these emerging options, the preparation of high-entropy PBA materials, the use of mechanochemistry to synthesize PBAs, and the use of high-throughput methodologies have been selected as highly promising emerging strategies to advance the field.

### 5.1 High-Entropy PBAs

In material science, the ’high-entropy’ term refers to systems containing at least five different metals, each ranging from five to thirty-five percent in atomic percentage.^166^ Initially, high-entropy metallic alloys were primarily utilized due to their enhanced mechanical properties compared to lower entropy (three or four metals) or binary alloys (Figure 7a).^167^ Over time, scientists realized that the benefits of high-entropy alloys extended beyond mechanical properties,^168,169^ leading to the emergence of a whole field dedicated to understanding these complex systems.

**Figure 7:**
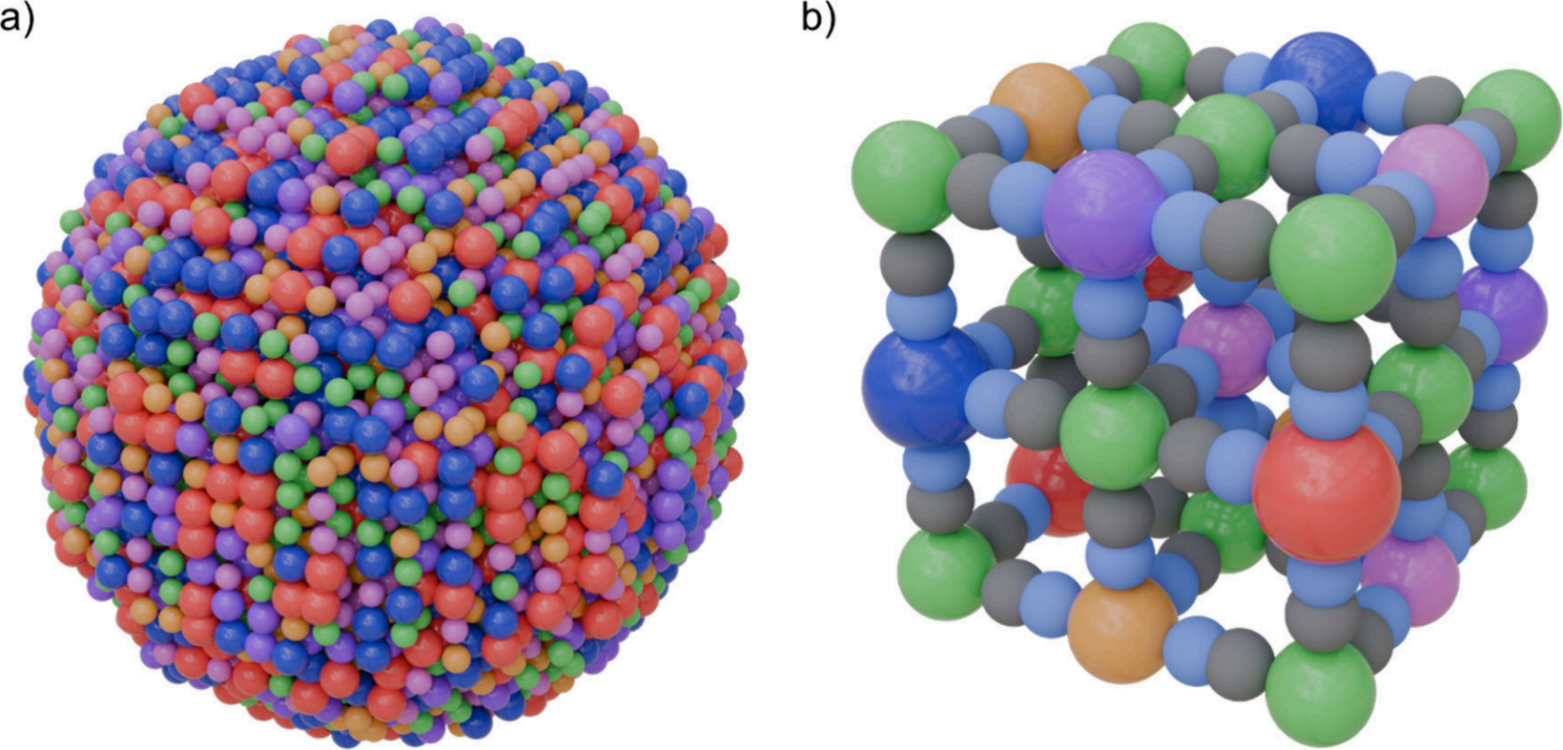
(a) High-entropy alloy, with different colors representing different metals; (b) T-site high-entropy PBA. The color code used is as follows: R-site (green), T-site (pink, dark blue, red, purple, and orange), C (gray), N (blue).

High-entropy materials exhibit numerous properties that make them highly attractive in the realm of functional materials.^170^ For instance, the ’high-entropy effect’, which arises from increased configurational entropy (i.e., the number of different metals), promotes the formation of solid solution structures. Additionally, the combination of metals of varying sizes and properties leads to inherent crystal lattice distortions and slower diffusion kinetics compared to pure and low-entropy alloys. The slower diffusion can be explained kinetically: a metal diffusing through a matrix of mixed metals encounters more barriers than it would in a single-metal matrix, resulting in reduced diffusion rates. Finally, high-entropy materials benefit from the ’cocktail effect,’ where synergistic effects emerge from the unique combination of different metal properties.^171^

In the case of PBAs, the high-entropy term usually refers to compounds presenting four or more different T-site cations with general formula A_*x*_T_*y*_T_w_T_*z*_T_r_T_v_[R­(CN)_6_] (T = Mg, Cr, Mn, Fe, Ni, Cu, Zn) (Figure 7b). In 2021, the first high-entropy PBA was isolated using a mechanochemical synthetic approach.^172^ The benefits of this approach will be discussed in the following mechanochemistry section, but the conclusion of this initial work was that the inclusion of metals never before introduced in PBAs had been possible. Moreover, the inclusion of some of these metals led to an increase in the capacitance of the final materials, as in the case of K_*x*_MgMnFeCoNi­[Fe­(CN)_6_] (metal ratios for Mg/Mn/Fe/Co/Ni were 0.1:0.25:1:0.27:0.29), which displayed a specific capacity of 175 F/g.

Following this work, high-entropy PBAs were first synthesized via solution synthesis using the Route 1 approach.^173^ The structural features of the obtained high-entropy Na_1.19_Fe_0.2_Mn_0.2_Ni_0.2_Cu_0.2_Co_0.2_[Fe­(CN)_6_]_0.79_·1.16H_2_O PBA and control medium-entropy PBAs (high-entropy minus one T-site cation at a time) were studied in detail using XAS to confirm the connectivity of the T- and R-site cations. Moreover, operando XRD analysis of the electrochemical extraction/insertion of Na ions from/into the high-entropy PBA revealed that the high configurational entropy enabled near-zero structural strain upon sodium de/insertion. This near-zero strain translated into extended cyclability of the material compared to medium entropy PBAs, retaining 93% of its capacity after 150 cycles, compared to 88% for the best of the medium entropy PBAs.^173^ Conversely, the presence of various metal cations in the structure is expected to have a significant influence on the interaction between the electrolyte anion and the PBA itself. However, both experimental and theoretical evidence are still lacking to support this hypothesis.

More works have followed the high-entropy PBA wave, some focusing on synthetic methodologies for controlling or tuning composition, particle size, and shape of the material.^174,175^ Others have focused on the low strain experienced by the framework to exploit the material in K-ion,^176^ Al-ion,^177^ or Zn-ion^178^ batteries. Nonetheless, all these works have been carried out on Fe-based PBA cathode materials. Thus, the proven enhanced stability of high-entropy PBA cathode materials is certainly a promising result to tackle the low stability of PBA anode materials. Preparing high-entropy anode Mn- or Cr-based PBA materials is foreseen to be one of the most promising strategies to widen the narrow library of stable PBA anode materials.

In addition to the research focused on stability, two possibilities seem to have been overlooked in high-entropy PBA materials: the tuning of the electrochemical redox potential of the R-site cation and the further reduction of the redox potential of known PBAs. Tuning can be achieved and controlled through the careful selection of T-site cations, as these directly influence the redox potential of the R-site cation.^9^ Additionally, the reduction of the redox potential in known PBAs can be accomplished by incorporating unconventional T-site cations — a feature observed exclusively in high-entropy PBAs, as in the case of Mg^2+^.^172^

Thus, in high-entropy PBAs, having synthetic control over the choice and atomic ratios of the T-site cations should enable the precise tuning of PBA redox potentials, facilitating the design of battery materials with specific voltage characteristics. Moreover, introducing unconventional T-site metals could further reduce the redox potential of known PBAs, opening up avenues for the development of PBA anode materials. This compositional control is expected to be a significant breakthrough in the advancement of PBAs as anode materials.

### 5.2 Mechanochemistry

The use of mechanochemical synthetic approaches is on the rise in several fields of chemistry, including pharmaceutical, organic, and inorganic synthesis.^72^ The earliest systematic investigations of mechanochemistry date back to the late 19th century, when mechanical force was applied using a mortar and pestle to crush and mix materials.^179^ Since then, mechanochemistry has evolved into a highly sophisticated technique. Today, solid-state chemical precursors are mixed in ceramic or metallic jars containing grinding balls, and high-speed shaking or rotation induces collisions between the balls, which are coated with the chemical precursors. These continuous collisions generate intense mechanical forces and localized heating, enabling chemical reactions that would otherwise require extreme conditions of pressure or temperature in solution.^72^

Initially developed for solvent-free reactions, particularly those requiring anhydrous conditions, mechanochemistry has demonstrated remarkable versatility. Solvent-assisted reactions, carried out in the micro- to milliliter range, have also yielded excellent results.^180^ Advances in characterization techniques have further expanded the scope of mechanochemistry, making it a cost-effective approach for the preparation of a wide range of functional materials.^181^ Among these materials, PBAs prepared using mechanochemical approaches have garnered significant interest.^172,182–184^

One key advantage of PBAs prepared via mechanochemistry is their low vacancy content, typically below 15%.^182,184^ Moreover, mechanochemical synthesis has been shown to effectively fill [R­(CN)_6_]_*y*_ vacancies by reacting vacancy-rich PBAs with their corresponding hexacyanometallate salts.^185^ For instance, in the case of K_0.1_Mn­[Co­(CN)_6_]_0.70_·*x*H_2_O PBA, mechanochemical reaction with K_3_Co^III^(CN)_6_ resulted in around 30% of the vacancies being filled, yielding a material with formula K_0.1_Mn­[Co­(CN)_6_]_0.80_·*x*H_2_O. Beyond vacancy control, mechanochemistry has enabled the incorporation of metals into the T-site that are otherwise difficult to introduce via conventional coprecipitation methods. A notable example is K_*x*_MgMnFeCoNi­[Fe­(CN)_6_], where Mg was successfully incorporated into the T-site for the first time, leading to a significant increase in capacity compared to similar materials lacking Mg.^172^ Although the precise mechanism behind T-site metal incorporation remains unclear, this capability represents a promising strategy for designing high-performance electrode materials. However, while mechanochemistry offers advantages in composition control, its inherently forceful nature limits control over particle size and shape.^72^

Beyond compositional tuning, mechanochemistry is particularly valuable for synthesizing materials whose precursors are unstable in aqueous environments. This is the case for Mn-based PBAs prepared via Route 1, where the instability of K_3_Mn^III^(CN)_6_ in water has historically hindered the exploration of these materials as anode candidates. Mechanochemistry provides a direct solution by enabling synthesis in a water-free environment while also allowing precise control over vacancy content.

Thus, mechanochemistry holds significant prospects to transform the development of PBA anode materials on multiple fronts. First, by correcting vacancy defects in Cr- and Mn-based PBAs, it can enhance conductivity and reduce polarization, improving ion insertion kinetics. Second, its ability to introduce underexplored metals into the T-site (or even the R-site) opens the door to PBAs with unprecedentedly low redox potentials, as demonstrated in *High-Entropy PBAs*. Finally, mechanochemistry enables the water-free synthesis of Mn-based PBAs, overcoming a long-standing challenge that has limited their development. By addressing these key barriers, mechanochemistry could play a pivotal role in advancing next-generation PBA anode materials for energy storage applications.

### 5.3 High-Throughput Methodologies

In the pursuit of material’s discovery, the constraints of time and manual labor, imposed by human involvement in conducting reactions and evaluating their outcomes, create notable bottlenecks.^186^ This challenge has become even more apparent with the discovery of high-entropy materials, where the vast number of possible compositions presents a daunting task for traditional synthetic and characterization methods.

To overcome this issue, high-throughput methodologies have been developed, enabling rapid material discovery while minimizing labor-intensive processes.^187^ In high-throughput synthesis, multiple reactions are conducted simultaneously with slight variations in conditions to assess their impact on the final material. For example, in a theoretical high-throughput solution-based synthesis, a series of seven high-entropy PBA anode materials with the general formula K_*x*_Mn_0.16_Fe_0.16_Co_0.16_Ni_0.16_Cu_0.16_ Zn_0.16_[Cr­(CN)_6_] could be prepared, systematically omitting one T-site cation at a time to study its effect. While traditional approaches could take several days to produce these materials, high-throughput synthesis enables their fabrication within a single day, dramatically improving efficiency. Beyond compositional tuning, reaction conditions such as the addition of acids or alkali salts can also be explored to refine material properties.

While synthesis is a crucial step, efficient characterization is essential to prevent bottlenecks in material discovery. Returning to the previous example, high-throughput characterization techniques such as ICP,^188^ powder XRD,^189^ and SEM^190^ provide rapid insights into composition, structure, and particle properties, facilitating subsequent electrochemical assessment. To meet growing scientific demands, modern analytical instruments have been adapted for high-throughput workflows, significantly expediting characterization. Furthermore, cost-effective, custom-built setups have been developed in-house, addressing the prohibitive expenses of commercial high-throughput systems.^190,191^

Following characterization, electrochemical evaluation is a pivotal yet challenging step, requiring large, homogeneous material samples. High-throughput electrochemical screening methodologies, such as scanning droplet cells (SDCs)^192,193^ and recessed microelectrodes,^194,195^ have been developed to streamline this process.

SDCs confine the electrochemical cell within a head-container that features a reference and counter electrode and a ∼ 1 mm aperture at the bottom. An electrolyte droplet dispensed through the aperture completes the electrochemical circuit upon contact with the working electrode surface. By scanning across a material library, SDCs generate electrochemical activity maps correlating composition with performance.^196,197^

Cavity microelectrodes, on the other hand, use minute sample quantities in confined cavities, resulting in reaction volumes of 10^–6^ to 10^–8^ cm^3^ — orders of magnitude smaller than conventional carbon composite electrodes (10^–1^ cm^3^).^198^ This dramatically reduces double-layer capacitance (*C* *_dl_*) and minimizes ohmic drop (*R* *_e_* · *I*; *R* *_e_* is the electrolyte resistance and *i* the current), enabling electrochemical cycling at scan rates in the hundreds of millivolts per second, compared to the tens of millivolts achievable with traditional electrodes.^199,200^ Consequently, battery materials can undergo ∼2,000 charge/discharge cycles within a week, whereas conventional testing could take weeks. This accelerated approach has been used to deconvolute diffusion limitations in PB-based nanoelectrodes for mediated redox-flow batteries.^201^ Additionally, recessed microelectrodes have recently been demonstrated for high-throughput screening of PBA cathodes and anodes under diverse electrolytes and high C-rates (up to 20C).^202^

For PBA anode materials, high-throughput methodologies offer a transformative path to accelerate material discovery and characterization — an area that has lagged behind PBA cathode development. On one hand, high-throughput synthesis can enable the rapid preparation of Cr-based PBAs, allowing systematic control over vacancy content and counterion incorporation to enhance cyclability. On the other hand, the ability to swiftly synthesize high-entropy PBAs would facilitate the incorporation of uncommon metals into the PBA framework, potentially lowering the operating redox potential of PBA anode materials and expanding their viability. High-throughput characterization will consolidate these advances by establishing rapid correlations between synthetic parameters and final material properties. Finally, high-throughput electrochemical screening via recessed microelectrodes represents a particularly promising approach for evaluating PBA anode materials with subtle compositional variations, as well as for simultaneously screening multiple electrolytes. This comprehensive strategy will not only optimize electrochemical properties but also enhance stability assessments across diverse electrolyte environments.

Thus, emerging high-throughput methodologies can significantly accelerate the discovery, characterization, and testing of PBA anode materials. However, the generation of vast amounts of characterization data must be accompanied by thoughtful interpretation. In this regard, rapid advancements in artificial intelligence (AI) and machine learning (ML) are playing a crucial role in materials discovery, enabling the efficient analysis of thousands of data points.^203,204^ AI and ML could be particularly transformative for PBA anode research, a field that requires substantial advancements to catch up not only with PBA cathodes but also with competing aqueous Na- and K-ion battery anodes, such as metal oxides and hard carbons. Nevertheless, it is essential to approach ML- and AI-driven data interpretation with a critical perspective, ensuring that conclusions remain grounded in the specific research context to avoid unrealistic claims.^205^

## 6 Open Questions

With the challenges set, the existence of useful past horizons, and the proposed emerging horizons, promising avenues for advancing the field of PBA anode materials have been shown. However, these promising approaches also bring forth several unresolved questions that require attention.

Initially, two main questions arise regarding the tuning of the composition of Cr-based PBAs. First, can the properties of Cr-based PBAs be fine-tuned similarly to those of Fe-based PBAs? Expanding on this inquiry, can the fine-tuning of the composition of PBA anode materials improve their stability during electrochemical cycling? These two questions are difficult to separate due to their intrinsic value. On one hand, refining the composition of PBAs has proven to be a crucial strategy for enhancing the electrochemical stability of certain cathode Fe-based PBAs.^56^ Although hexacyanochromate precursor salts do not seem to impose any restrictions in terms of chemical stability and reactivity, conclusive answers regarding the degree to which the properties of Cr-based PBAs can be fine-tuned through modifications of the synthetic conditions can only be obtained experimentally. On the other hand, only the synthesis of these PBA anode materials with fine-tuned properties will shed light on whether enhanced stability can be achieved with this approach.

The application of coatings of varying compositions on PBA anode materials also prompts several unanswered questions. Can the coating process for PBA anode materials be carried out similarly to that for PBA cathode materials? And will these coatings enhance the stability of PBA anode materials, as observed in PBA cathode materials? Within the realm of PBAs, only PBA cathode materials have been subjected to coating with different polymeric materials, which reportedly improve conductivity, chemical stability, and cyclability.^162,164,165^ Furthermore, the cost-effectiveness of this method makes it highly appealing for mitigating stability-related limitations in PBA anode materials. Therefore, addressing these inquiries is crucial for advancing the field and similarly to the previous open questions related to composition tuning in Cr-based PBAs, only experimental work can shed light on its feasibility.

The emergence of high-entropy PBA cathode materials raises crucial questions in the field of PBA anode materials. First, is the synthesis of high-entropy PBA anode materials feasible? Initially appearing straightforward, this question gains complexity when considering the challenges associated with precursor chemistry and the limited synthetic exploration for Mn- and Cr-based PBAs. The instability of K_3_Mn^III^(CN)_6_ in aqueous solutions^98,99^ imposes constraints on experimental conditions, possibly requiring the synthesis of high-entropy Mn-based PBAs through mechanochemistry. Conversely, although the more stable K_3_Cr^III^(CN)_6_ permits certain synthetic modifications in aqueous solutions,^96^ the lack of exploration in binary Cr-based PBAs introduces uncertainty about the feasibility of preparing high-entropy Cr-based PBAs.

The second and third questions concern the extent to which the observed enhanced stability of PBA cathode materials will translate to PBA anode materials. Regarding ligand isomerization in PBA anode materials, can high configurational entropy stabilize ligands and prevent isomerism? Since ligand isomerism is an energy-activated process,^206^ the sluggish diffusion provided by high configurational entropy should have an important effect in lowering isomerization degrees, likely inhibiting it completely.^171^ This question is pivotal for advancing the field of PBA anode materials, as isomerization constraints further limit available PBA anode material options.

Regarding the stability of high-entropy PBA anode materials during electrochemical cycling, can high configurational entropy improve the high solubility problem in PBA anode materials? As noted earlier, the formation of stable T···An*^ads^* complexes upon electrochemical cycling and the structural strain induced by the distortion of the PBA bonds are critical factors leading to the dissolution of PBAs.^139^ The demonstration of near-zero structural strain in high-entropy PBA cathode materials establishes a precedent for enhanced structural stability during electrochemical cycling.^173^ Determining whether high configurational entropy inhibits or reduces the formation of the T···An*^ads^* complexes due to the presence of metals with distinct chemical properties is a fundamental question. Understanding this phenomenon would provide insights into whether the near-zero structural strain observed in PBA cathode materials can be replicated in PBA anode materials. Both experimental and theoretical evidence are necessary to address these questions.

## 7 Wrap up

We began this perspective by properly framing the applicability of PBA anode materials, addressing a crucial question: *why should research in this direction be pursued?* Establishing this rationale is a necessary step toward their development for next-generation batteries. As discussed earlier, PBAs are unlikely to compete with anodes used in portable electronics due to economic and practical limitations. However, they hold significant promise for alternative applications, particularly in stationary and grid-scale intercalation-based aqueous Na- or K-ion batteries, where established anode options are lacking. The unsuitability of carbon derivatives (HC, graphite) in aqueous Na- and K-ion batteries leaves NaTi_2_(PO_4_)_3_ and KTi_2_(PO_4_)_3_ as the only primary options — a limited selection that poses significant bottlenecks for further development. In this context, PBAs emerge as a compelling alternative: they are cost-effective, exhibit long-term cycling stability, and support high rate capabilities — key attributes for grid-scale applications. This capability is already being recognized, with companies like Natron Energy and Sharp investing in the development of all-PBA batteries.^9^

With this foundation established, we explored the fundamental characteristics of PBAs that make them attractive as electrode materials, such as their open framework and chemical tunability. This led us to a second key question: *why has research on PBA anode materials remained so limited?*

Several factors likely contribute to this underexploration. One possibility is that the research community has not yet fully recognized the appropriate framing of these materials. While other studies have outlined their strengths and limitations, no dedicated perspective has focused specifically on PBA anodes — perhaps reflecting their perceived limited applicability. Additionally, the overwhelming focus on Li-ion batteries, combined with the unsuitability of PBAs as anodes in that context, may have shaped research priorities away from this direction.

Beyond framing, technical challenges have also played a role. Many PBA anodes may have underperformed electrochemically due to stability issues, leading to unreported failures. Competition from established anodes such as metal phosphates and oxides has also limited visibility. Moreover, PBA anodes tend to perform best when paired with PBA cathodes due to their similar kinetics and cycling stability, making them less attractive for standalone anode research.^9^ Finally, it remains an open question whether PBAs are fundamentally better suited as cathode materials rather than anodes, which would explain the significant disparity in research attention between PBA anode and cathode materials. Likely, a combination of these factors has hindered progress in the field.

Importantly, many of these challenges are not unique to PBAs. For instance, NaTi_2_(PO_4_)_3_ — the current benchmark anode for aqueous Na-ion anodes — also suffers from limited electronic conductivity, rate capability, and cycling stability.^15,16,207^ Yet, despite these drawbacks, it remains the most viable option.^10,11^ In light of this, and given the chemical versatility of PBAs, it is surprising that they have not been more thoroughly investigated as aqueous anodes — especially considering the extensive work on PBA cathodes and related materials.

To address this imbalance, the second part of this perspective focused on identifying the challenges facing PBA anode materials and outlining a roadmap for advancing their development. We highlighted key synthetic and postsynthetic strategies to enhance their performance, stability, and scalability. These approaches not only open promising research directions but also raise fundamental questions that must be addressed.

In conclusion, while research on PBA anode materials has been slow to gain momentum, this very lack of exploration presents a wealth of opportunities for innovation. By leveraging their exotic characteristics and emerging methodologies, researchers can address open questions, redefine the role of PBA-based anodes, and unlock their full capabilities for next-generation energy storage solutions.

## Abbreviations

AI: Artificial intelligence
DLS: dynamic light scattering
EDX: energy dispersive X-ray measurements
EELS: electron energy loss spectroscopy
FTIR: Fourier transform infrared spectroscopy
HC: Hard carbon
HER: Hydrogen evolution reaction
ICP: Inductive coupled plasma-mass spectroscopy
ML: Machine learning
OER: Oxygen evolution reaction
PB: Prussian Blue
PBA: Prussian Blue Analogues
PEDOT: Poly­(3,4-ethylenedioxythiophene)
PSS: polystyrenesulfonate
PVP: Polyvinylpyrrolidone
SAXS: Small-angle X-ray scattering
SEM: Scanning electron microscopy
SFC: Scanning flow cell
sXAS: Soft X-ray absorption spectroscopy
TEM: Transmission electron microscopy
TGA: Thermogravimetric analysis
XAS: X-ray absorption spectroscopy
XPS: X-ray photoelectron spectroscopy
XRD: X-ray diffraction
